# Meta-analysis of genome-wide association studies for loin muscle area and loin muscle depth in two Duroc pig populations

**DOI:** 10.1371/journal.pone.0218263

**Published:** 2019-06-12

**Authors:** Zhanwei Zhuang, Shaoyun Li, Rongrong Ding, Ming Yang, Enqin Zheng, Huaqiang Yang, Ting Gu, Zheng Xu, Gengyuan Cai, Zhenfang Wu, Jie Yang

**Affiliations:** 1 College of Animal Science and National Engineering Research Center for Breeding Swine Industry, South China Agricultural University, Guangdong, P.R. China; 2 National Engineering Research Center for Breeding Swine Industry, Guangdong Wens Foodstuffs Group Co., Ltd, Guangdong, P.R. China; Universita degli Studi di Bologna, ITALY

## Abstract

Loin muscle area (LMA) and loin muscle depth (LMD) are important traits influencing the production performance of breeding pigs. However, the genetic architecture of these two traits is still poorly understood. To discern the genetic architecture of LMA and LMD, a material consisting of 6043 Duroc pigs belonging to two populations with different genetic backgrounds was collected and applied in genome-wide association studies (GWAS) with a genome-wide distributed panel of 50K single nucleotide polymorphisms (SNPs). To improve the power of detection for common SNPs, we conducted a meta-analysis in these two pig populations and uncovered additional significant SNPs. As a result, we identified 75 significant SNPs for LMA and LMD on SSC6, 7, 12, 16, and 18. Among them, 25 common SNPs were associated with LMA and LMD. One pleiotropic quantitative trait locus (QTL), which was located on SSC7 with a 283 kb interval, was identified to affect LMA and LMD. Marker ALGA0040260 is a key SNP for this QTL, explained 1.77% and 2.48% of the phenotypic variance for LMA and LMD, respectively. Another genetic region on SSC16 (709 kb) was detected and displayed prominent association with LMA and the peak SNP, WU_10.2_16_35829257, contributed 1.83% of the phenotypic variance for LMA. Further bioinformatics analysis determined eight promising candidate genes (*GCLC*, *GPX8*, *DAXX*, *FGF21*, *TAF11*, *SPDEF*, *NUDT3*, and *PACSIN1*) with functions in glutathione metabolism, adipose and muscle tissues development and lipid metabolism. This study provides the first GWAS for the LMA and LMD of Duroc breed to analyze the underlying genetic variants through a large sample size. The findings further advance our understanding and help elucidate the genetic architecture of LMA, LMD and growth-related traits in pigs.

## Background

The pig, which has abundant phenotypes, is a model animal that not only intimately resembles man in physiology, anatomy, and genetic architecture[1] but also contributes to meat consumption[2]. In the past decades, lean pigs account for a large share of the pork consumption market, and the traits that affect swine growth have been important breeding targets in the pig industry[2]. Loin muscle area (LMA) and loin muscle depth (LMD) play important roles in the determination of growth traits (e.g., back fat and carcass lean rate)[3]. Thus, prediction of growth traits by investigating LMA and LMD is important. It is generally accepted that both of nutrition and growth environment of the pig can affect the growth and development of loin muscle[4, 5], and muscle growth may be subject to number of muscle fibers in swine muscle composition[6]. However, genetics is widely considered to be the most important factor for the loin muscle development. Hence, it is the primary means to achieve this goal in terms of genetics, such as finding causal genes or mutations that affect muscle development[7, 8]. LMA and LMD are heritable and moderate-heritability above traits, where the estimated heritabilities range from 0.35 to 0.47[9–11], showing that these two traits can be improved via genetic approach.

To dissect the molecular basis of the divergent phenotypes of LMA and LMD among different individuals, researchers have selected various populations[12–14] using multiple strategies[15, 16]. For instance, using exiguous microsatellite markers across the pig entire genome, genome scans have detected a limited number of quantitative trait loci (QTLs) for LMA[17] and LMD[18]. Despite some progress in the traditional genetic improvement of carcass and growth-related traits, inadequacies and challenges remain to be overcome to elucidate the biological mechanisms of complex traits[19].

To date, 27,878 QTLs associated with 718 traits have been reported in the pig QTL database (http://www.animalgenome.org/cgi-bin/QTLdb/SS/index, Jul 24, 2018). Among them, 330 and 41 QTLs are associated with LMA and LMD, respectively. These discoveries have provided a certain number of molecular markers to the breeding for swine LMA and LMD. For instance, Edwards et al.[20] used 510 Duroc × Pietrain F2 animals genotyped for 124 microsatellite markers that are uniformly spaced across the genome and detected a significant QTL that affects LMA and related backfat trait. A similar approach was employed in a study related to LMD[21]. The researchers detected a significant QTL on *Sus scrofa* chromosome (SSC) 11 that explains approximately 6% of the phenotypic variance of LMD. Although many QTLs have been detected, these findings are still deficient due to the inferior power of QTL mapping and poor resolution in the majority of QTLs[22]. Another factor that cannot be ignored is that the confidence intervals are commonly in the order of ~30 cM, and such a large region may contain several genes, which hamper the optimization of plausible candidate genes[23, 24]. With the development of genomic tools and high-density single nucleotide polymorphism (SNP) marker panels in recent years, genome-wide association studies (GWAS), a powerful strategy in detecting genetic variants associated with complex traits, have been widely used in humans[25], and livestock[26, 27]. In pigs, GWAS have been conducted to explore the genetic variants and genetic architecture on a great diversity of importantly economic traits[28]. These studies successfully identified causal mutation and verified the causal genes[29]. For LMA and LMD, GWAS have also been performed to detect the underlying genetic mechanisms[4, 30], but the sample size of these studies is smaller compared with that of the current study. Although GWAS have obtained remarkable findings[31], the use of association studies in the genetics of human and other species is bound by population structure and limited sample size[32]. Therefore, to enhance the detection power and verify the significant discoveries identified in single-population GWAS, meta-analysis of GWAS was widely used in human[33] and livestock[34] owing to the powerful statistical efficiency.

In this study, we performed a GWAS, based on phenotype records using Porcine SNP50 Beadchip in two Duroc pig populations with different genetic contexts (American origin and Canadian origin). The American and Canadian original populations comprised 3916 and 2127 individuals, respectively. Moreover, a meta-analysis combining the full GWAS results from the two populations was conducted and uncovered additional significant SNPs. This study aimed to identify genomic regions and plausible candidate genes that lead to the phenotypic diversity of LMA and LMD. This study can serve as a basis to elucidate the molecular architecture underlying muscle development in pigs, humans, and other mammals.

## Materials and methods

### Ethics statement

All procedures involving animals in this study met the guidelines for the care and use of experimental animals established by the Ministry of Agriculture of China. The ethics committee of South China Agriculture University (SCAU) (Guangzhou, China) approved this study (Approval number SCAU#0017).

### Animals and phenotype

In this study, experimental animals, including American and Canadian origin Duroc populations, were raised in the Wens Foodstuff Group Co., Ltd. (Guangdong, China) following the same feeding standards. Briefly, a total of 6043 pigs of these two Duroc populations were used in the present study, including 3916 American origin pigs and 2127 Canadian origin pigs. The pigs of the American original population were born between 2013 and 2017, and the other was born between 2015 and 2017. All pigs of the two populations sustained uniform feeding conditions and fine fodder and consistent management during the fattening period from 30 to 100 kg live weight to minimize the impact of non-genetic factors. Phenotypic records included LMA and LMD. For American and Canadian populations, phenotypes of LMA and LMD were collected by practiced investigators from the 10th-rib to 11th-rib of pigs at the weight of 100 ± 5 kg by an Aloka 500V SSD B ultrasound (Corometrics Medical Systems, USA), which employed diagnostic ultrasound system and transducers to acquire high resolution images, and computer software was used to ascertain the LMA[11].

### Genotyping and quality control

Genomic DNA was extracted from ear tissue following the traditional phenol/chloroform method. The quality and quantity of the DNA samples were measured with a NanoDropTM 2000 (Thermo Fisher Scientific, Waltham, MA, USA) as described by Wang et al.[35]. In brief, all DNA samples were assessed by ratios of light absorption (A260/A280) in the range of 1.8–2.0 and diluted to a final concentration of 50ng/μL. Genotyping was conducted using Geneseek Porcine 50K SNP chip (Neogen, Lincoln, NE, United States), which contains 50,703 SNP markers across the entire genome. Quality control (QC) procedures were carried out using PLINK v1.07 software[36] with the parameters of animal call rates > 0.95, SNP call rates > 0.9, minor allele frequencies > 0.01, and *P* > 10^−6^ for Hardy–Weinberg equilibrium test. SNPs with no position information and located on the sex chromosomes were also removed. Notably, the two populations follow the same quality control criteria. After QC, a final set of 38,790 and 35,845 informative SNPs and 3916 and 2127 individuals were retained for single-trait GWAS in the American and Canadian original Duroc populations, respectively. Furthermore, in the meta-analysis of GWAS, a common set of 40,030 differently eligible SNPs for LMA and LMD across the populations were later used.

### Population structure analysis

In consideration of the different genetic contexts of the two Duroc populations, principal component analysis (PCA) was performed to detect the possible population stratification using GCTA tool[37]. We initially conducted a blended PCA by combing the two populations with the first two eigenvectors belonging to each population and then drew a PCA plot. The top five eigenvectors were embedded subsequently as covariates in the association analysis model to correct and decrease the confounding effect of population structure in the massive sample association study herein[38]. Meanwhile, genomic control, a prevailing method for solving stratification, was also performed to acquire the genomic inflation factors (λ). Then, a quantile-quantile (Q-Q) plot was constructed using R script to assess the influence of population stratification on the GWAS.

### Single-trait GWAS

The entire processes in the single-trait GWAS were performed using the software of genome-wide efficient mixed-model Analysis (GEMMA)[39] and genome-wide complex trait analysis (GCTA)[37]. The genomic relatedness matrix between individuals within each population was used to perform single-trait GWAS by GEMMA. GEMMA provides two alternative options (the centered genotypes or standardized genotypes) to estimate the relatedness matrix from genotypes. Although the choice of which way to use relies on the underlying genetic mechanism of a given trait, the two matrices exert negligible effects[39]. In the present study, the standardized option (“-gk 2”) was used to estimate a n × n relatedness matrix. GWAS was performed independently for LMA and LMD in each Duroc pig population with a univariate linear mixed model fitted in GEMMA. The estimated n × n standardized relatedness matrices (K) between the individuals within populations as well as the calculated top five eigenvectors were used in the model as follows:

y=Wα+Xβ+u+ε

where y is the vector of phenotypic values; W is the incidence matrix of covariates (fixed effects) that comprise the top five eigenvectors derived from PCA, sex and live weight; α is the vector of corresponding coefficients including the intercept; X is the vector of marker genotypes; β is the corresponding effect size of the marker; u is an m × 1 vector of random effects, with u ~ MVNm(0, λ τ^−1^K); and ε is the vector of random residuals, with ε ~ MVNn(0, τ^−1^In). τ^−1^ is the variance of the residual errors; λ refers to the ratio between the two variance components; K is a known n × n relatedness matrix calculated in previous step; and I refers to the identity matrix. MVNn denotes the n-dimensional multivariate normal distribution. Bonferroni correction was applied to determine the genome-wide and chromosome-wide significance thresholds, which were defined as 0.05/N and 1/N, respectively. N is the number of filtered SNPs for each trait of American and Canadian original Duroc populations.

### Meta-analysis

A meta-analysis was performed across the different genetic contexts of Duroc pig populations for a trait on the strength of single-trait analysis using METAL software[40]. METAL converts the direction of effect and the P value observed from each study ί into Z-scores. The key statistic formulae for the approach used herein was previously described[40, 41]. Notably, 40,030 common SNPs were detected in the American and Canadian origin populations. Bonferroni correction was applied to determine the genome-wide significance threshold (0.05/40,030) and the chromosome-wide significance threshold (1/40,030).

### SNP-based heritability estimation and contribution to phenotypic variance

The GCTA software was used to estimate the variance explained by genome-wide SNPs (SNP-based heritability) for each trait in the two populations via the restricted maximum likelihood approach, relying on the genomic relationship matrix estimated from all the autosomal SNPs. To partition the phenotypic variance onto each of the autosomes, we also appointed a model to investigate the contributions by each chromosome as described by Yang et al.[37]. Furthermore, the GCTA tool was used to calculate the phenotypic variance explained by each suggestive or significant SNP, which was detected by single-trait GWAS. Notably, the fixed effects including the first five PCA eigenvectors, sex and live weight were embedded as covariates to adjust the population stratification and latent relatedness[42].

### LD analysis and conditional analysis

Many SNPs, which were located on several narrow regions, were associated with LMA and LMD in the Canadian original Duroc population, and they were detected both by single-trait and meta-analysis GWAS. To further verify the linkage disequilibrium among these SNPs and detect candidate regions connected with the two traits, we employed PLINK v1.07[36] and Haploview v4.2[43] for haplotype block analysis. We also performed conditional analysis to determine the potential associated SNPs that might be masked in the putative region by intense signal. In brief, the peak SNP or the one that explained the largest phenotypic variance was selected to perform a univariate linear mixed model via converting its genotypes into covariates[28, 44]. Keeping that in mind, the other covariates used previously were consistent. Regional association plots were generated using the R package v3.4.3.

### Candidate gene search and functional annotation

The functional genes were searched on the strength of Sscrofa 11.1 genome version (http://asia.ensembl.org/Sus_scrofa/Info/Index). Gene sets with the criteria of including or nearest the significant SNPs were selected to conduct KEGG pathways and Gene Ontology (GO) analysis. Pathway analysis and GO analysis was conducted using the DAVID bioinformatics resource (https://david.ncifcrf.gov/, Aug 6, 2018, version 6.8). Fisher’s exact test was used to assess the significance of the enriched terms with P < 0.05 and detect the genes involved in biological processes[2, 45]. In addition, GeneCards (http://www.genecards.org/) and the NCBI (https://www.ncbi.nlm.nih.gov/) database were used to query gene functions and determine promising candidates.

## Results

### Phenotype and heritability analysis

We acquired four phenotypic data from two Duroc populations of 6043 pigs, which met the quality control standards, including LMA and LMD. Descriptive statistics of phenotypes and the SNP-based heritability of LMA and LMD are shown in Table 1. The coefficients of variation (CV) of LMA and LMD in the American and Canadian original populations ranged from 7.10% to 8.94% and 7.89% to 11.22%, respectively. Results showed that different scopes of CV values indicated different levels of sample variability between the two pig populations. The SNP-based heritability of each trait was estimated using a univariate model, where the phenotypic variance was partitioned into the variance explained by genetic components and residual variance with the genetic relationship matrix. The genomic heritabilities of the traits in the two populations are moderate-heritability, ranging from 0.37 to 0.39, showing that genetic technology could effectively promote the genetic improvement of these traits.

**Table 1:** Descriptive statistics and heritability for loin muscle area (LMA) and loin muscle depth (LMD) traits in two Duroc populations.

Traits^1^	Source	Unit	N	Mean (±SD)	Min	Max	C.V./%	h^2^ (±SE)
LMA	American origin	cm^2^	3916	42.28±3.78	26.40	54.80	8.94	0.37±0.02
LMD		mm	3916	52.36±3.72	37.30	65.00	7.10	0.39±0.02
LMA	Canada origin	cm^2^	2127	39.14±4.39	27.30	56.60	11.22	0.37±0.03
LMD		mm	2127	47.91±3.78	37.00	61.20	7.89	0.38±0.03

Number (N), Mean (standard deviation), Minimum (Min), Maximum (Max), coefficient of variation (C.V.), heritability (standard error) of LMA and LMD traits values.

^1^Loin muscle area (LMA); Loin muscle depth (LMD)

### Single-trait GWAS

**Fig 1:**
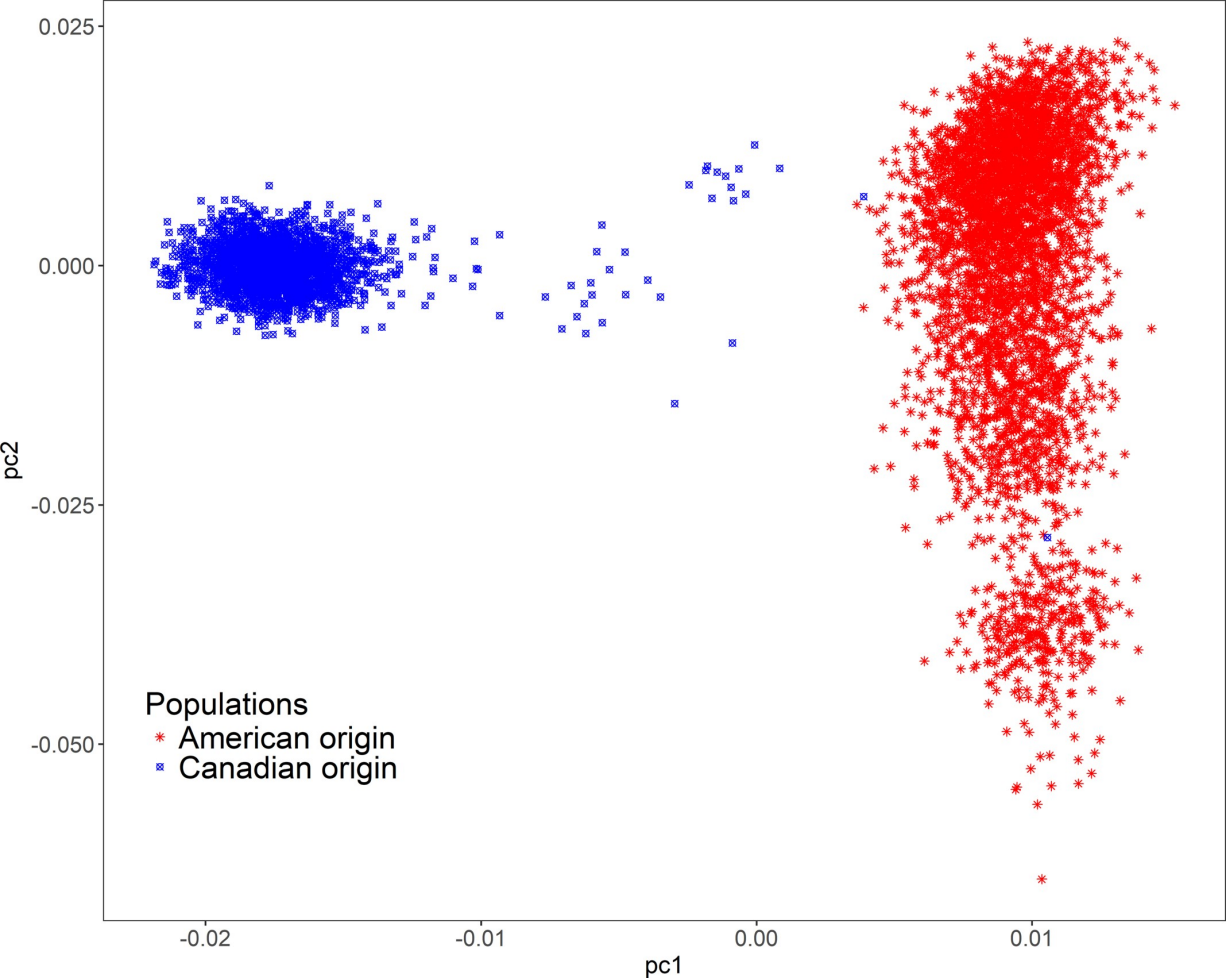
Population structure for two Duroc populations. pc1 = first principal component; pc2 = second principal component. The first two PCs derived from the genomic kinship matrix were extracted to assess the population structure. In this study, the two Duroc pig populations can be clearly identified via principal component analysis.

**Fig 2:**
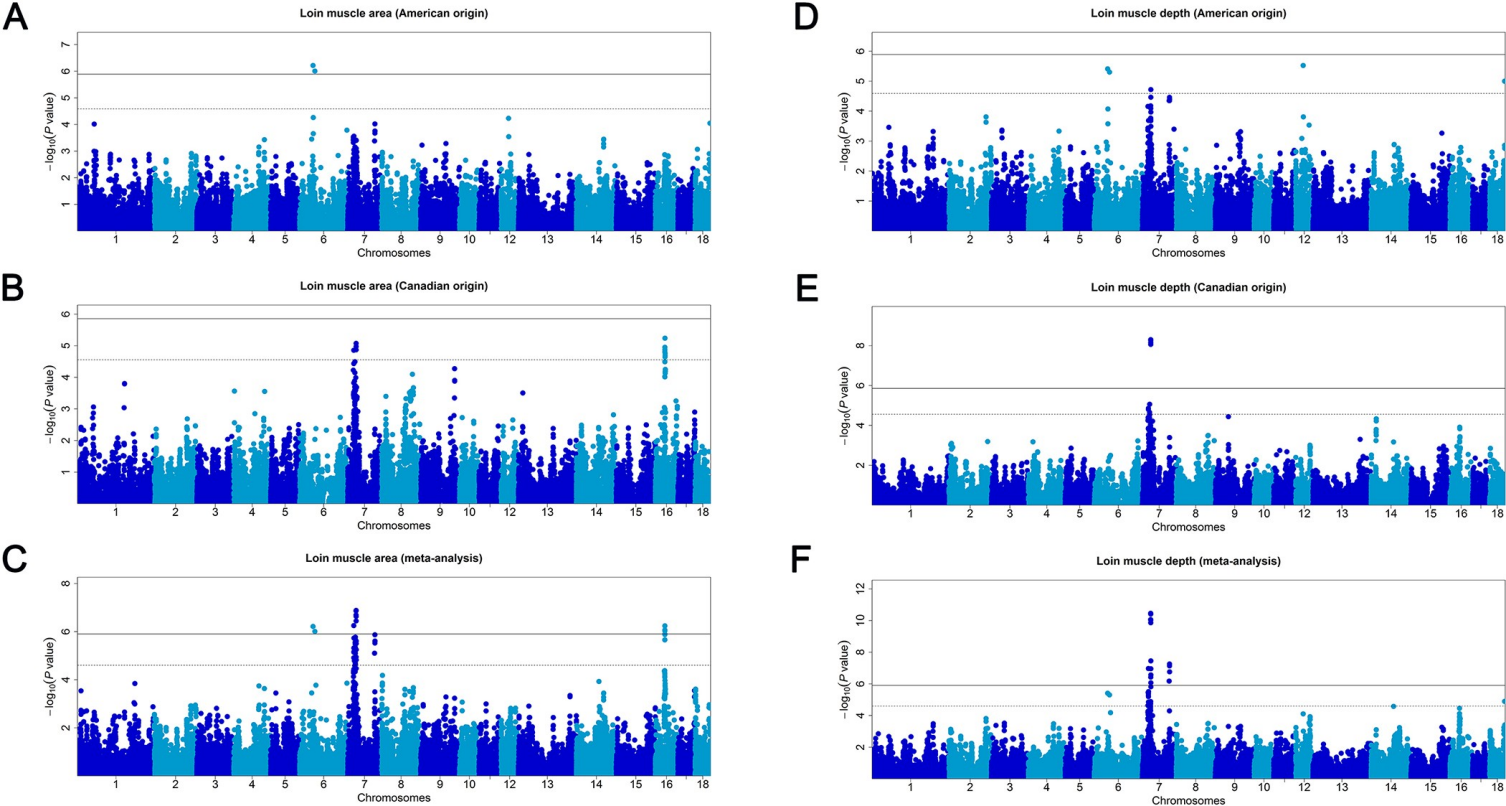
Manhattan plots of genome-wide association studies for LMA and LMD in the two Duroc pig populations. The x-axis represents the chromosomes, and the y-axis represents the -log10(*P*-value). Different colors indicate various chromosomes. The solid and dashed lines indicate the 5% genome-wide and chromosome-wide Bonferroni corrected thresholds, respectively. The thresholds of the genome-wide and the chromosome-wide levels are *P* < 1.29E-06 and *P* < 2.58E-05 (American population); *P* < 1.39E-06 and *P* < 2.79E-05 (Canadian population); *P* < 1.25E-06 and *P* < 2.50E-05 (meta-analysis). Manhattan plots on the left are shown for the single-trait association analysis in (A) American original population, (B) Canadian original population, and (C) meta-analysis for LMA. Manhattan plots on the right are shown for the single-trait association analysis in (D) American original population, (E) Canadian original population, and (F) meta-analysis for LMD.

The effects of potential population stratification were corrected using PCA. In this study, the two Duroc pig populations were clearly identified via PCA, as shown in Fig 1. Although almost all individuals were classified into one cluster within each population, the plot implied a slight population stratification. The Manhattan and QQ plots for LMA and LMD are shown in Fig 2 and S1 Fig. The genomic inflation factor (λ) at each trait ranged from 1.03 to 1.10, and none of the Q-Q plots showed any sign of inflation, indicating consistent consequence with PCA.

We herein performed four separate association analyses for LMA and LMD in the two populations using a univariate linear mixed model. Multiple significant SNPs associated with LMA and LMD for the American and Canadian original populations of Duroc breed are listed in Tables 2 and 3, respectively. In summary, 24 SNPs were detected on SSC6, 7, 12, 16, and 18. Notably, eight SNPs showed significant association with more than one trait, suggesting that they exert pleiotropic effects on multiple carcass traits.

**Table 2:** Significant SNP and associated genes for LMA in the single-trait association analysis.

SSC^1^	SNP ID	Position(bp)^2^	*P* value^3^	Population	Minor/major Allele	MAF^4^	EPV/%^5^	Candidate gene	Distance/bp^6^
6	Hal_2	47357966	**6.05E-07**	American	T/C	0.094	1.18	*RASGRP4*	24726
	12784654	54079560	**9.84E-07**	American	T/C	0.094	1.18	*FGF21*	-3046
7	ALGA0040260	30342161	8.40E-06	Canadian	A/G	0.216	1.77	*NUDT3*	-1092
	ALGA0040263	30356985	8.48E-06	Canadian	G/A	0.216	1.77	*NUDT3*	within
	ASGA0032536	30476054	1.06E-05	Canadian	C/A	0.217	1.74	*PACSIN1*	within
	ASGA0032526	30497305	1.06E-05	Canadian	C/T	0.217	1.74	*PACSIN1*	within
	INRA0024788	30317219	1.35E-05	Canadian	T/C	0.217	1.72	*ENSSSCG00000032242*	-3195
	DRGA0007345	21886880	1.39E-05	Canadian	G/A	0.362	1.74	*ENSSSCG00000032646*	474
16	WU_10.2_16_35829257	33757844	5.75E-06	Canadian	C/A	0.328	1.83	*SNX18*	-18902
	ASGA0072998	33589982	1.12E-05	Canadian	T/C	0.328	1.81	*ARL15*	24383
	ALGA0090190	33515233	1.34E-05	Canadian	A/G	0.328	1.78	*ARL15*	within
	ALGA0090184	33467573	1.35E-05	Canadian	G/A	0.328	1.78	*ARL15*	within
	MARC0103451	33493718	1.35E-05	Canadian	T/C	0.328	1.78	*ARL15*	within
	MARC0074818	33559933	1.35E-05	Canadian	T/C	0.328	1.78	*ARL15*	within
	ASGA0073002	33636702	1.57E-05	Canadian	T/G	0.328	1.79	*ARL15*	71103
	ALGA0090276	34334858	1.76E-05	Canadian	A/G	0.303	1.79	*GPX8*	-650
	ALGA0090273	34364519	2.07E-05	Canadian	A/G	0.303	1.78	*MCIDAS*	-13441
	ALGA0090242	34031872	2.26E-05	Canadian	C/T	0.303	1.78	*ENSSSCG00000016898*	-9810

^1^Sus scrofa chromosome.

^2^SNP position in Ensembl.

^3^The bold data in this column represent the significant SNP surpass the genome-wide significant threshold, otherwise at the chromosome-wide significant level.

^4^Minor allelic frequency.

^5^The proportion of the phenotypic variance explained by significant SNP.

^6^The location of SNP in upstream/downstream of the nearest gene.

**Table 3:** Significant SNP and associated genes for LMD in the single-trait analysis.

SSC^1^	SNP ID	Position(bp)^2^	*P* value^3^	Population	Minor/major Allele	MAF^4^	EPV/%^5^	Candidate gene	Distance/bp^6^
6	Hal	47357966	3.88E-06	American	T/C	0.094	1.04	*RASGRP4*	24726
	12784654	54079560	4.97E-06	American	T/C	0.094	1.05	*FGF21*	-3046
7	MARC0061142	30716800	1.91E-05	American	T/C	0.158	0.61	*SNRPC*	within
12	ALGA0065784	26376192	2.98E-06	American	T/C	0.27	1.2	*ENSSSCG00000034682*	-555
18	WU_10.2_18_58542037	53382393	9.98E-06	American	T/C	0.165	0.59	*INHBA*	477180
7	ALGA0040263	30356985	**4.96E-09**	Canadian	G/A	0.216	2.48	*NUDT3*	within
	ALGA0040260	30342161	**5.47E-09**	Canadian	A/G	0.216	2.48	*NUDT3*	-1092
	ASGA0032536	30476054	**7.05E-09**	Canadian	C/A	0.217	2.45	*PACSIN1*	within
	ASGA0032526	30497305	**7.05E-09**	Canadian	C/T	0.217	2.45	*PACSIN1*	within
	INRA0024788	30317219	**8.60E-09**	Canadian	T/C	0.217	2.43	*ENSSSCG00000032242*	-3195
	DRGA0007345	21886880	1.39E-05	Canadian	T/C	0.105	2.05	*ENSSSCG00000032646*	474
	ALGA0039866	26451150	8.72E-06	Canadian	G/A	0.362	2.15	*TINAG*	within
	ALGA0039447	21940478	1.57E-05	Canadian	T/C	0.361	1.98	*ZNF165*	within
	M1GA0027226	26125115	2.21E-05	Canadian	A/G	0.274	1.67	*FAM83B*	within

^1^Sus scrofa chromosome.

^2^SNP position in Ensembl.

^3^The bold data in this column represent the significant SNP surpass the genome-wide significant threshold, otherwise at the chromosome-wide significant level.

^4^Minor allelic frequency.

^5^The proportion of the phenotypic variance explained by significant SNP.

^6^The location of SNP in upstream/downstream of the nearest gene.

Five SNPs were significantly associated with LMA and LMD in the American original Duroc population, of which two common SNPs were associated with LMA and LMD at different significance levels (Tables 2 and 3). These two SNPs were located close to genes RAS guanyl releasing protein 4 (*RASGRP4*) (SNP on SSC6: 47.35Mb) and fibroblast growth factor 21 (*FGF21*) (SNP on SSC6:54.07Mb). They both explained 1.18% of the phenotypic variance of LMA. Three SNPs were associated with LMD at the chromosome-wide significance level. Of these SNPs, the most significant was ALGA0065784 (SSC12: 26.37Mb) and was located close to gene ENSSSCG00000034682, and it explained 1.20% of the phenotypic variance of LMD.

For the Canadian original Duroc pig population, 19 SNPs were associated with LMA and LMD. As shown in Tables 2 and 3, six common SNPs were associated with these two traits at different significance levels. Among them, six SNPs associated with LMA reached the chromosome-wide significance level (*P* < 2.79E-05), and five SNPs associated LMD reached the genome-wide significance level (*P* < 1.39E-06). The most significant SNP, ALGA0040260, was located at 30.34Mb and was near the gene Nudix hydrolase 3 (*NUDT3*) on SSC7. This SNP explained 1.77% of the phenotypic variance of LMA. As for LMD, the peak SNP in this region, ALGA0040263, was located at 30.35 Mb within the *NUDT3* gene on SSC7 and contributed 2.48% of the phenotypic variance of LMD. The remaining 13 SNPs reached the chromosome-wide significance level, of which 10 were associated with LMA (all located on SSC16) and three with LMD (all located on SSC7). The most significant SNP of the 10 SNPs, WU_10.2_16_35829257 contributed 1.83% of the phenotypic variance of LMA. This SNP was not located within any gene; nonetheless, it was in close proximity to gene sorting nexin 18 (*SNX18*). In addition, the remaining SNPs, which were close to each other on SSC16 between 33.46 Mb and 34.37 Mb, were located within or near several genes, such as ADP ribosylation factor like GTPase 15 (*ARL15*), glutathione peroxidase 8 (putative) (*GPX8*), and multiciliate differentiation and DNA synthesis associated cell cycle protein (*MCIDAS*), and a not openly reported gene (*ENSSSCG00000016898*) (Table 2).

### Meta-analysis across populations by trait

We conducted a meta-analysis of GWAS for each trait across these two Duroc pig populations. The meta-analysis integrates all association signals from the two populations. Thus, when the two populations show consistent association directions with the traits of interest, the detection power for these traits improve via the meta-analysis. In the current study, 68 SNPs associated with LMA and LMD were identified in the across-population meta-analysis. Among them, 56 SNPs were associated with LMA, and 11 of these 56 SNPs reached the genome-wide significance level (*P* < 1.25E-06). In addition, 37 SNPs were associated with LMD, and 15 of these 37 SNPs reached the genome-wide significance level (*P* < 1.25E-06) (Fig 2; S1 Table). Among these 68 SNPs, 25 common SNPs were associated with LMA and LMD simultaneously. In addition to all the significant SNPs, 17 significant SNPs that were detected in the single-trait association analysis were confirmed and 51 loci with novel candidate genes were identified in the meta-analysis. To evaluate whether SNPs associated with LMA and LMD traits in present study replicate any previously known QTLs, we searched the pigQTLdb based on SNP locations (S2 Table). Although a large number of QTLs that were mainly detected via QTL mapping using sparse microsatellite markers were identified for LMA and LMD in multiple pig breeds, few QTLs were reported within a narrow interval using the GWAS strategy. Compared with previous results, our findings revealed many loci in several confined genetic regions and highlighted two QTLs that influence LMA and LMD.

### Haplotype block analysis and conditional analysis

**Fig 3:**
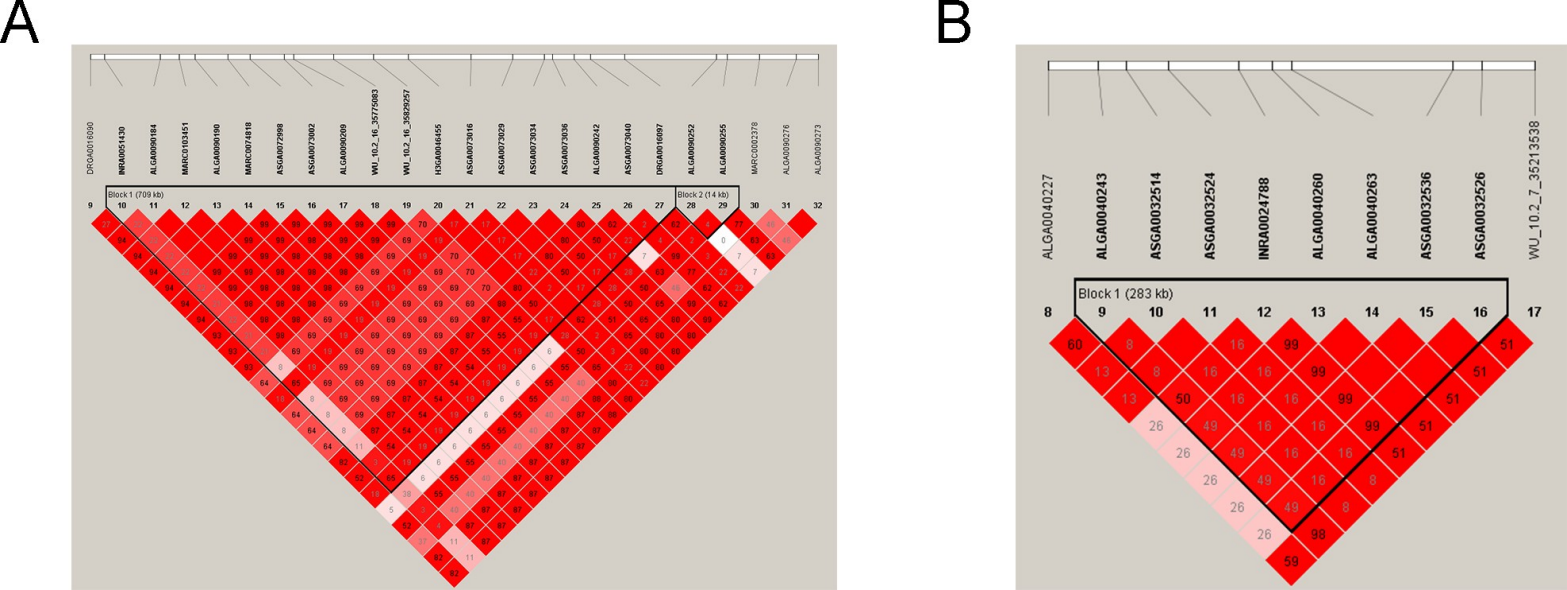
**Linkage disequilibrium (LD) blocks in the significant region on SSC16 (A) and SSC7. (B).** LD blocks are marked with triangles. Values in boxes are LD (r^2^) between SNP pairs and the boxes are colored according to the standard Haploview color scheme. The complete red boxes with no numbers indicated that SNP pairs have complete linkage disequilibrium. Annotated genes in the chromosomal region were retrieved from the Ensemble genome browser (www.ensembl.org/Sus_scrofa/Info/Index).

**Fig 4:**
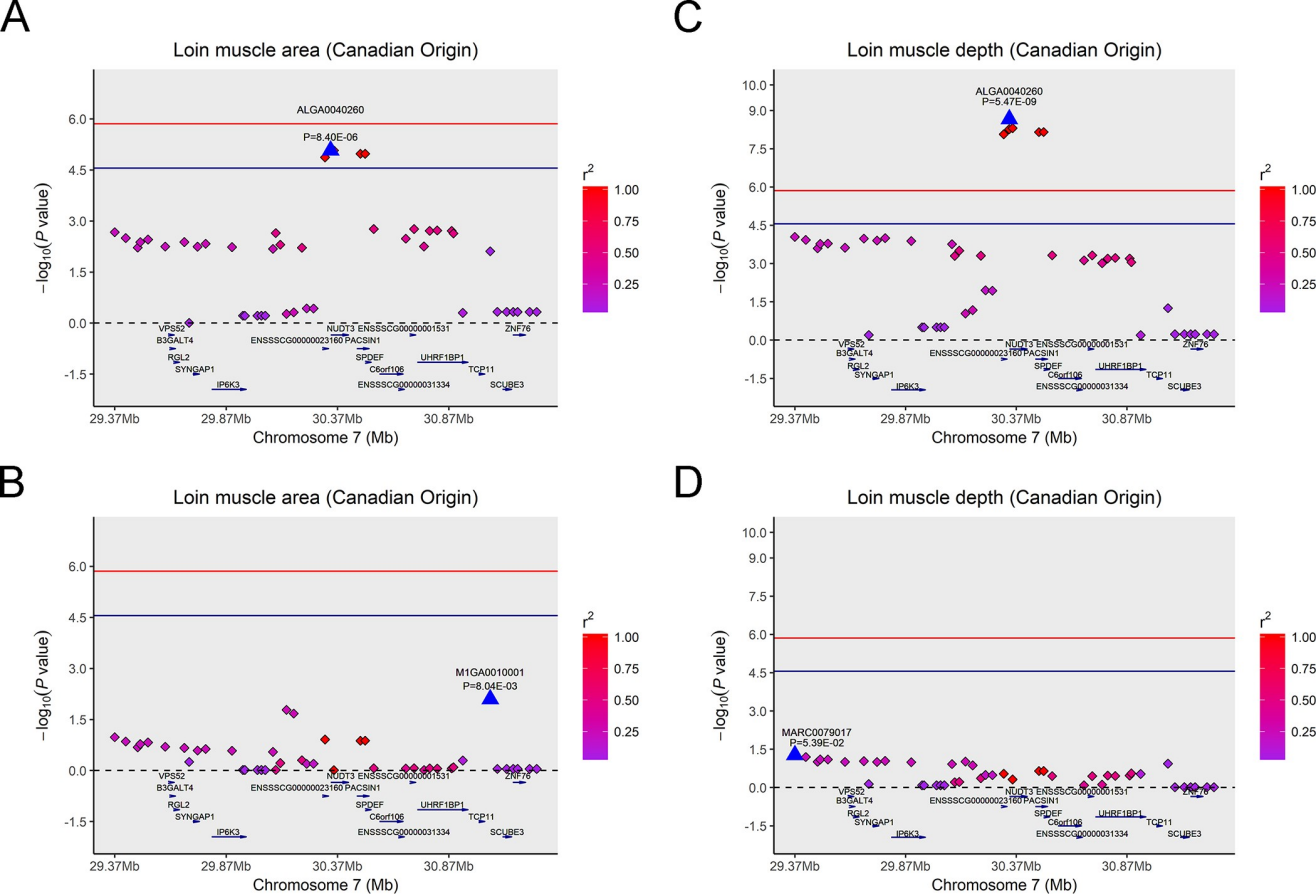
Regional association plot of the primary signal (ALGA0040260) associated with LMA and LMD at SSC7. For each plot, the -log10(observed *P*-values) of SNPs (y-axis) are presented according to their chromosomal position (x-axis). The red line and darkblue line indicate the genome-wide significance level (*P* < 1.39E-06) and the chromosome-wide significance level (*P* < 2.79E-05), respectively. The primary SNPs are denoted by large blue triangles. SNPs are represented by colored rhombi according to the target SNP with which they were in strongest LD. The left panel of the figure shows the association results for LMA (A) before and (B) after conditional analysis on ALGA0040260. The right panel of the figure shows the association results for LMD (C) before and (D) after conditional analysis on ALGA0040260. The P-value of association results for (B) LMA and (D) LMD after conditional analysis on ALGA0040260 fell below the predicted threshold.

**Fig 5:**
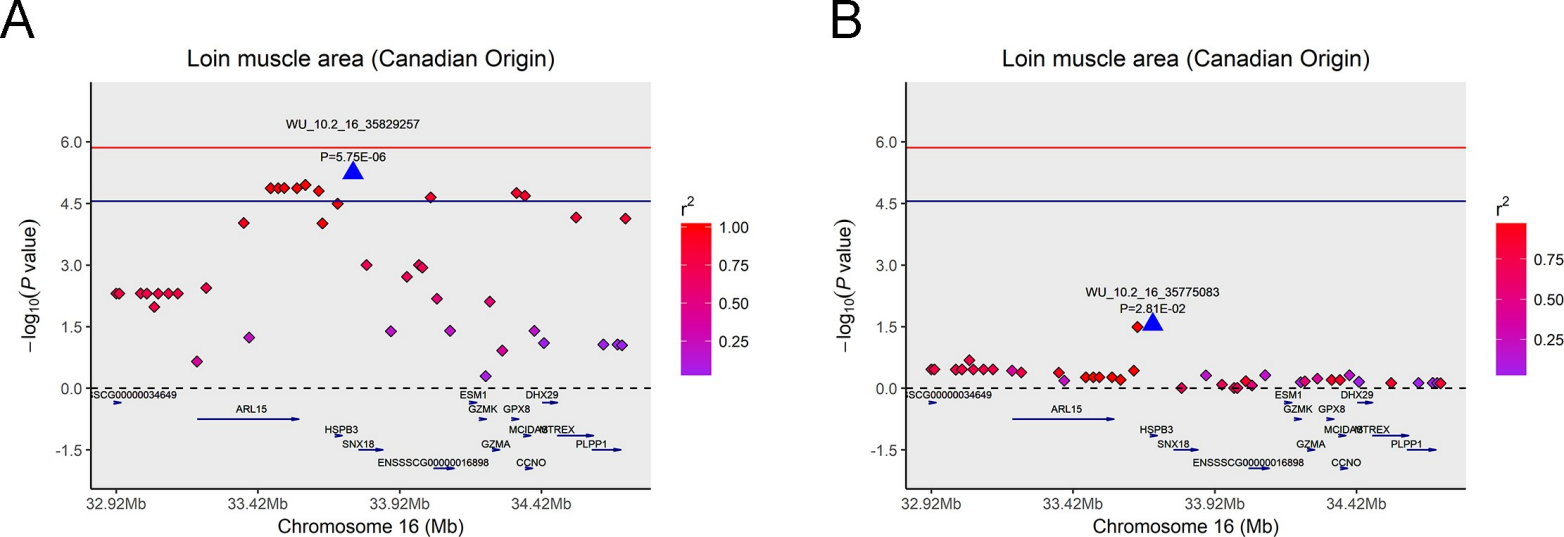
Regional association plot of the primary signal (WU_10.2_16_35829257) associated with LMA at SSC16. For each plot, the -log10(observed *P*-values) of SNPs (y-axis) are presented according to their chromosomal position (x-axis). The red line and darkblue line indicate the genome-wide significance level (*P* < 1.39E-06) and the chromosome-wide significance level (*P* < 2.79E-05), respectively. The primary SNPs are denoted by large blue triangles. SNPs are represented by colored rhombi according to the target SNP with which they were in strongest LD. The left panel of the figure shows the association results for LMA (A) before conditional analysis on ALGA0040260. In the right panel of the figure (B), the P-value of corresponding SNPs fell below the predicted threshold.

As mentioned above, among the 19 significant SNPs in the Canadian original population, we found eight significant SNPs on SSC16 in close proximity to be associated with LMA and detected a haplotype block that spanned 709 kb (Fig 3A) containing the eight SNPs. In particular, we observed five significant SNPs associated with LMA and LMD, and these significant SNPs were situated in a 283 kb block on SSC7 (Fig 3B). To examine whether linkage disequilibrium (LD) masks certain independently associated SNPs and/or potential signals, we performed a step-wise conditional analysis. Then we fitted the peak SNP ALGA0040260 on SSC7 that was significantly associated with LMA and LMD in the Canadian original population into the univariate linear mixed model as a covariate to inspect these associations. For LMA, the P values of previous significant SNPs that had strong LD status with lead SNP ALGA0040260 decreased below the minimum threshold line (Fig 4A and 4B). The same pattern was found for the same peak SNP for LMD trait (Fig 4C and 4D). Analogously, the peak SNP WU_10.2_16_35829257 on SSC16 that was merely significantly associated with LMA in the Canadian original population was also fitted into the model as a covariate in the same manner. The same pattern was also observed as shown in Fig 5A and 5B.

### SNP effects and partitioning phenotypic variance by chromosomes

**Fig 6:**
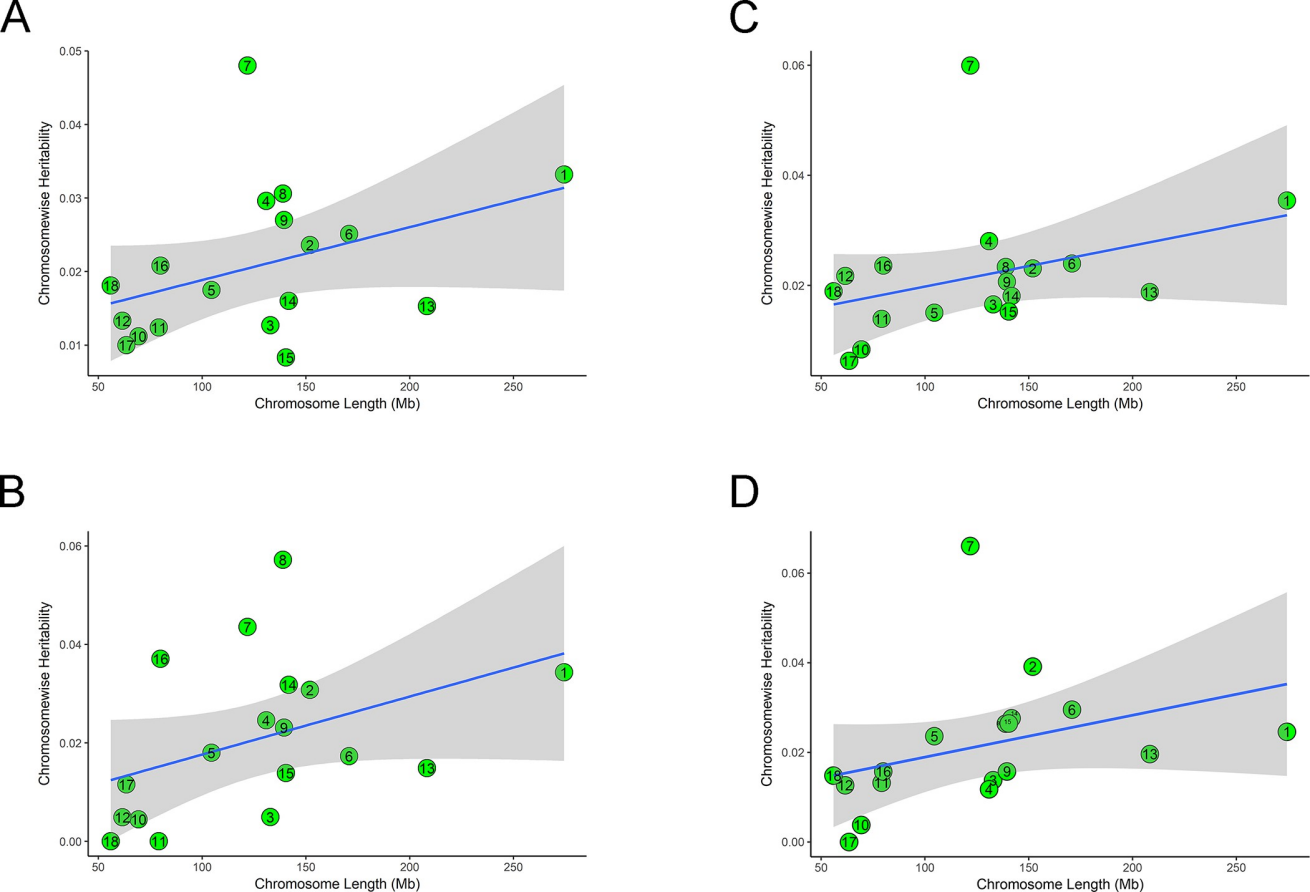
Heritabilities of LMA and LMD by chromosome in the two populations. Estimates of chromosome-wide heritability on LMA and LMD are drawn against the chromosome length (x-axis). The blue line represents heritability regressed on chromosome length. Grey area around the blue line is the 95% confidence level interval for prediction from the linear model. In the figure, (A) and (B) show the heritability that each chromosome explained for LMA in the American and Canadian original populations, respectively. (C) and (D) show the heritability that each chromosome explained for LMD in the American and Canadian original populations, respectively.

The phenotypic variance explained by significant SNPs, which were detected by single-trait association analysis, and autosomes were estimated for LMA and LMD in the two populations using GCTA tool[37] (Tables 2 and 3, Fig 6). For LMA, the peak SNP, Hal_2, located on SSC6, accounted for 1.18% of the phenotypic variance in the American original population. However, the significant SNPs in the Canadian original population accounted for 1.72%-1.83% of the phenotypic variance, and the lead SNP, WU_10.2_16_35829257, located on SSC16, had the largest contribution (1.83%) to the phenotypic variance. For LMD, the significant SNPs in the American original population explained 0.59%-1.20% of the phenotypic variance. The most significant SNP, ALGA0065784, located on SSC12, accounted for 1.20% of the phenotypic variance. In the Canadian original population, nine significant SNPs explained 1.67%-2.48% of the phenotypic variance. Notably, the peak SNP, ALGA0040263, and the second most significant SNP, ALGA0040260, made an equal contribution to the phenotypic variance (2.48%), and they were located on a narrow region on SSC7 between 30.34 and 30.35 Mb.

The phenotypic variance explained by each chromosome for LMA and LMD is shown in Fig 6. The contribution of autosomes differed from each other, and much new information was obtained. For instance, chromosomes SSC7, SSC8, and SSC16 accounted for 4.35%, 5.72%, and 3.71% of LMA variance in the Canadian original population, respectively. Although the contribution of SSC8 explained the largest fraction of LMA variance, single-trait association analysis or meta-analysis did not detect any significantly associated SNPs on this chromosome. Meanwhile, single-trait association analysis identified numerous SNPs significantly associated with LMD on SSC7 in the same population, and chromosome 7 explained approximately 6.61% of LMD variance. These findings are in line with the results of association analysis. The peak shown in Fig 6A and 6C implied that SSC7 should have identified a large number of significant SNPs, whereas few SNPs were detected on this chromosome in the American original population.

### Candidate genes and function analysis

A total of 41 functional genes that were within or nearest the significant SNPs were detected based on the Sus scrofa 11.1 genome assembly (Tables 2 and 3 and S1 Table). These genes were used to conduct KEGG pathways and Gene Ontology (GO) analysis. In particular, the gene set enrichment analysis uncovered that 21 annotated genes had a highlight biology function with LMA and LMD (S3 Table). In brief, the most significant pathways for LMA and LMD were related to glutathione metabolism and MAPK signaling pathway, respectively. The top GO term for LMA was related to transcription and DNA templated whereas that for LMD was related to regulation of transcription and DNA templated. Given that numerous genes are involved in important pathways and biological processes, functional annotations in the GeneCards tools and NCBI database as well as copious literatures were investigated and verified. Hence, eight genes, including Glutamate-Cysteine Ligase Catalytic Subunit (*GCLC*), *GPX8*, death domain associated protein (*DAXX*), fibroblast growth factor 21 (*FGF21*), TATA-Box binding protein associated factor 11 (*TAF11*), SAM pointed domain containing ETS transcription factor (*SPDEF*), *NUDT3*, and protein kinase C and casein kinase substrate in neurons 1 (*PACSIN1*), with biological functions such as increasing available amino acids in skeletal muscle, mammalian growth, fat deposition, and body size of pig were selected as promising candidates for swine carcass traits and growth-related traits.

## Discussion

### QTL associated with LMA and LMD

Scientific and comprehensive determining indices are the basis of measuring the level of production performance and growth-related traits of swine. LMA and LMD are important traits influencing the production performance of commercial pigs. Thus, the genetic mechanisms of LMA and LMD should be elucidated to estimate the affecting factors and consider them for future pig breeding programs. In this study, we performed four single-trait GWAS in two Duroc pig populations containing 6043 individuals in total. To improve the power of detection for common SNPs, we conducted a meta-analysis combining the full GWAS results from the two populations and uncovered additional significant SNPs. To our knowledge, few studies used GWAS strategy to analyze LMA and LMD. The present study is the first GWAS for LMA and LMD focusing on Duroc breeding pigs. Based on a large sample size (n = 6043) and multiple statistical analysis strategies, a total of 75 SNPs associated with LMA and LMD were detected. Of these SNPs, only one third of the significant SNPs were found to be simultaneously associated with both LMA and LMD, although these two traits were highly positively correlated to each other in American (r = 0.94) and Canadian populations (r = 0.74). The partial overlap of significant SNPs between LMA and LMD may be because the muscle growth is not only depended on the muscle fiber density, but also the fiber diameter[6, 46, 47]. It means that the muscle fiber density and the fiber diameter may have great contribution to the development of loin muscle and consequently affect LMA and LMD. One QTL with pleiotropic effects on LMA and LMD on SSC7 was identified in our study. LD analysis uncovered one haplotype block of 283 kb, in which the most significant SNPs (ALGA0040260 and ALGA0040263) on SSC7 were associated with LMA and LMD in the Canadian original pig population, whereas a region that partially overlapped with the results herein was identified significantly associated with carcass length as described by Liu et al.[48]. Notably, previous studies demonstrated that a hot region that spans approximately 6.5 Mb (from 31.27Mb to 37.74Mb) on SSC7 exert strong QTL effects on carcass traits[14, 49]. Compared with previous studies, our findings indicated that the pleiotropic QTL for LMA and LMD was fine mapped within a narrow interval of 283 kb. In addition, on SSC16, a QTL that affects LMA was refined to a 709 kb interval in this study. In this QTL region, Cho et al.[12] identified a QTL for LMA at the more distal position from our QTL in an F2 intercross between Landrace and Korean native pigs (n = 830). In the same region, Choi et al.[13] reported a QTL for LMA that spanned from 24.3 cM to 44.8 cM using 954 F2 Duroc × Pietrain pigs. Our study increased the power of QTL detection and narrowed the QTL locations by using high-density molecular markers and large sample sizes. However, no common SNPs and QTL that were associated with the traits were shared with the two pig populations in this study. The results showed that the difference in genetic background greatly influenced the identification of significant SNPs. In subsequent studies, we will continue to increase the number of samples and the density of breed-specific markers or use re-sequencing data for the traits analyzed herein.

### Population stratification control in different genetic backgrounds

Given their powerful detection capability, GWAS are widely adopted for studying the genetics of natural variation. However, the most basic problem of GWAS is the risk of false positive in structured populations that may cause spurious associations[50, 51]. In the present study, three methods were used to resolve this problem: genomic control[28], PC analysis[38], and a linear mixed model that includes genomic relatedness matrix among individuals[52]. In the linear mixed model, the top five eigenvectors that calculated separately in the two different populations via GCTA tool[37] were embedded as covariates in the association analysis model to reduce the influence of population sub-stratification. As illustrated in S1 Fig, the Q-Q plot implies that the population stratification was sufficiently controlled with the aid of relatedness matrix and principal components of the specific population. Nevertheless, only a few variants were detected in the American original population. Most traits of interest are multifactorial and many factors affect the detection power of statistical of genetics studies[52]. In this study, we selected two Duroc pig populations with different backgrounds to perform the same analysis. The qualified sample size of the American original population is much larger than that of the Canadian original population (3196 versus 2127). Theoretically, the larger the sample size, the more genetic variations detected. However, a notable exception was detected. The probable reason is that rare variant SNPs have lower r2 compared to the common alleles contained in commercial SNP arrays because these SNPs are selected to represent common variants[52]. Thus, additional breed-specific SNP arrays should be developed to boost more dense representative tag variations for association analysis.

### Candidate genes

Basing from the results of KEGG pathway and GO analysis, we obtained a number of functional genes and performed further gene annotations. Eight genes related to LMA and LMD were selected as promising candidates, of which, two were relevant to LMA, one was relevant to LMD, and five were involved in both traits. For LMA, *GCLC* and *GPX8* are highlighted as important candidate genes that play roles in the pathway of glutathione metabolism. *GCLC*, the first rate-limiting enzyme of glutathione synthesis, is related to the biological processes of protein heterodimerization activity and coenzyme binding. Wei et al.[53] found that *GCLC* participates in amino acid metabolism and might upregulate the expression of glutathione in porcine skeletal muscle via feeding pigs with a linseed-enriched diet. *GPX8* functions in glutathione metabolism and thyroid hormone synthesis pathways. Damon et al.[54] discovered that *GPX8* is highly expressed in the longissimus muscle of Large White pigs with the function of response to oxidative stress and oxidation reduction. It was also identified by genome-wide linkage analysis as a candidate gene for growth and carcass merit in an F2 pig population because expression QTL (eQTL) analysis of loin muscle tissue demonstrated that *GPX8* eQTL is part of a network relevant to cell cycle and lipid metabolism[55]. For LMD, one candidate gene, *DAXX*, was related to LMD trait. *DAXX* participates in the MAPK signaling pathway and is also involved in the regulation of transcription and DNA templated. The *DAXX* gene encodes a multifunctional protein and interacts with various proteins, such as apoptosis antigen Fas. Yang et al.[56] found that *DAXX*, as a novel signaling protein, plays a critical role in Fas-binding domain, and the elevated expression of *DAXX* cements Fas-mediated apoptosis.

For LMA and LMD, many common significant SNPs that are involved in both traits were found to be close to or within numerous genes. Consequently, five genes were determined as major functional candidates: *FGF21*, *TAF11*, *SPDEF*, *NUDT3*, and *PACSIN1*. The *FGF21* gene encodes a member of the fibroblast growth factor (*FGF*) family, which plays a critical role in lipid metabolism[57], cell growth and development, and wound healing[58]. Ayuso et al.[59] found that *FGF21* is relevant to adipose and muscle tissue development, and functions in lipid metabolism in purebred Iberian pigs and in cellular and muscle growth in Duroc-crossbred Iberian pigs. *FGF21* also induces PGG-1α and regulates carbohydrate and fatty acid metabolism[60]. *TAF11* participates in RNA polymerase II transcription initiation and promoter clearance pathway and is related to the regulation of transcription and DNA templated. Robinson et al.[61] showed that TAF11 enhances TBP-TFIIA complex formation through protein-protein interactions, and the loss of interaction would encumber the cell growth and transcription in vivo. *NUDT3* of the Nudix protein family and participates in insulin signaling and inositol phosphate metabolism pathway. Many studies proved that *NUDT3* is the obesity-linked gene for human[62], Drosophila, and mouse[63], possibly because of its polymorphic activity against polyphosphate substrates[64]. *SPDEF* plays a role in DNA binding transcription factor activity, transcription, and DNA templated. Meanwhile, *PACSIN1* is involved in phospholipid binding and cytoskeletal protein binding. Baranski et al.[65] found that *SPDEF* and *PACSIN1* are associated with adiposity and the amount of fat/triglyceride in tissue-specific knocked down Drosophila. *NUDT3*, *SPDEF*, and *PACSIN1* have been identified in a closely linked region that is associated with obesity or carcass traits, such as body growth and size, in human or pigs[48, 66, 67]. These candidate genes warrant further investigation.

### Comparing the single-trait analysis with meta-analysis

In the present study, we performed four single-trait GWAS in American and Canadian original Duroc pig populations using the same linear mixed model and carried out a meta-analysis to improve the detection power. Comprehensive use of multiple methods improves the effectiveness of detection, but no common significant SNPs were identified between the two populations. For this case, some issues are worth discussing. First, significant SNPs detected by single-trait association analysis were significantly less than those detected by meta-analysis. The results suggest that the number of samples used in the experiment needs to be improved, although it far exceeds the number in a vast majority of previous studies; population specificity also possibly contributes to the occurrence of this event[31, 40]. Some informative SNPs that had large effects on the single-trait GWAS were eliminated during QC in the meta-analysis. Another intuitive difference between single-trait association analysis and meta-analysis is that the new P values for all significant SNPs were decreased, and numerous SNPs that were detected by single-trait association analysis were confirmed via meta-analysis. Similar findings have been discovered in previous studies that closely related to carcass traits. For instance, Jiang et al.[68] performed GWAS separately using American and British original Yorkshire populations to uncover significant variants and candidate genes related to growth and fatness traits, but they detected no overlapping significant SNPs in the two populations with different genetic backgrounds. Guo et al.[69] used four pig populations (a White Duroc × Erhualian F2 intercross population, Chinese Sutai, Laiwu, and Erhualian populations) and four methods (SS-GWAS, MS-GWAS, SM-GWAS, and MM-GWAS, the detailed introductions are shown in the article) to conduct an association analysis on nine growth and fatness traits. Results revealed no common loci across the four populations in the single-population GWAS. However, the meta-analysis was more powerful in detecting the association between markers and diverse phenotypes, and greater SNPs were perceived compared with the single-population association analysis. Despite is strong detection effectiveness, the meta-analysis might deduce a high false positive rate[40, 68]. Therefore, we focused on the overlapping SNPs of single-trait association analysis and meta-analysis, and conducted a subsequent functional analysis based on them.

## Conclusions

In sum, we detected a set of significant SNPs for LMA and LMD in 6043 Duroc pigs from two populations with different genetic contexts. One pleiotropic QTL located on SSC7 with a 283 kb interval was identified to affect multiple traits. Another genetic region on SSC16 (709 kb) was also discovered to play an important role in LMA. A series of bioinformatics analysis strategies revealed many functional genes for LMA and LMD. For instance, *GCLC*, *GPX8*, *DAXX*, *FGF21*, *TAF11*, *SPDEF*, *NUDT3*, and *PACSIN1* with biological functions such as increasing available amino acids in skeletal muscle, mammalian growth, fat deposition, and body size of pig were selected as promising candidates for swine carcass and growth-related traits. This study provides the first GWAS for the LMA and LMD of Duroc breed to analyze the underlying genetic variants through a large sample size. These findings revealed the complexity of the genetic mechanism that forms the phenotypic diversity and provide essential insights into the future production of pigs in the context of marker-assisted selection.

## Supporting information

S1 Fig Quantile–quantile (Q–Q) plots of genome-wide association studies for LMA and LMD in the two populations. Q–Q plots show the observed versus expected negative log10 P-values. The left panel of the figure shows Q–Q plots for LMA in (A) the American original population and (B) the Canadian original population, respectively. The right panel of the figure shows Q–Q plots for LMD in (C) the American original population and (D) the Canadian original population, respectively.
(TIF)

S1 Table Significant SNPs and associated genes for LMA and LMD in the meta-analysis. (DOCX)

S2 Table Comparative mapping of tag SNPs with previous QTLs reported in the pig QTL database (as of September 26, 2018) and previous GWAS results. (DOCX)

S3 Table Significant KEGG pathway and GO terms with loin muscle area (LMA) and loin muscle depth (LMD) (P-value<0.05). (XLSX)

## References

[1] Bendixen E, Danielsen M, Larsen K, Bendixen C. Advances in porcine genomics and proteomics-a toolbox for developing the pig as a model organism for molecular biomedical research. Brief Funct Genomics. 2010;9(3):208–19. 10.1093/bfgp/elq004 WOS:000278232900003. 20495211

[2] Wang K, Liu D, Hernandez-Sanchez J, Chen J, Liu C, Wu Z, et al Genome Wide Association Analysis Reveals New Production Trait Genes in a Male Duroc Population. PLoS One. 2015;10(9):e0139207 10.1371/journal.pone.0139207 26418247 PMC4587933

[3] Friesen KG, Nelssen JL, Goodband RD, Tokach MD, Unruh JA, Kropf DH, et al The effect of dietary lysine on growth, carcass composition, and lipid metabolism in high-lean growth gilts fed from 72 to 136 kilograms. J Anim Sci. 1995;73(11):3392–401. 10.2527/1995.73113392x . 8586599

[4] He Y, Ma J, Zhang F, Hou L, Chen H, Guo Y, et al Multi-breed genome-wide association study reveals heterogeneous loci associated with loin eye area in pigs. J Appl Genet. 2016;57(4):511–8. 10.1007/s13353-016-0351-8 . 27183999

[5] Yang XR, Yu B, Mao XB, Zheng P, He J, Yu J, et al Lean and obese pig breeds exhibit differences in prenatal gene expression profiles of muscle development. Animal. 2015;9(1):28–34. 10.1017/S1751731114002316 . 25229314

[6] Dwyer CM, Fletcher JM, Stickland NC. Muscle cellularity and postnatal growth in the pig. J Anim Sci. 1993;71(12):3339–43. Epub 1993/12/01. 10.2527/1993.71123339x . 8294285

[7] Van Laere AS, Nguyen M, Braunschweig M, Nezer C, Collette C, Moreau L, et al A regulatory mutation in IGF2 causes a major QTL effect on muscle growth in the pig. Nature. 2003;425(6960):832–6. 10.1038/nature02064 . 14574411

[8] Younis S, Schonke M, Massart J, Hjortebjerg R, Sundstrom E, Gustafson U, et al The ZBED6-IGF2 axis has a major effect on growth of skeletal muscle and internal organs in placental mammals. Proceedings of the National Academy of Sciences of the United States of America. 2018;115(9):E2048–e57. Epub 2018/02/15. 10.1073/pnas.1719278115 29440408 PMC5834713

[9] Godinho RM, Bergsma R, Silva FF, Sevillano CA, Knol EF, Lopes MS, et al Genetic correlations between feed efficiency traits, and growth performance and carcass traits in purebred and crossbred pigs. Journal of Animal Science. 2018;96(3):817–29. 10.1093/jas/skx011 WOS:000429789400004. 29378008 PMC6093586

[10] Kuhlers DL, Nadarajah K, Jungst SB, Anderson BL. Genetic selection for real-time ultrasound loin eye area in a closed line of Landrace pigs. Livest Prod Sci. 2002;72(3):225–31.

[11] Suzuki K, Kadowaki H, Shibata T, Uchida H, Nishida A. Selection for daily gain, loin-eye area, backfat thickness and intramuscular fat based on desired gains over seven generations of Duroc pigs. Livest Prod Sci. 2005;97(2–3):193–202. 10.1016/j.livprodsci.2005.04.007 WOS:000233689000012.

[12] Cho IC, Yoo CK, Lee JB, Jung EJ, Han SH, Lee SS, et al Genome-wide QTL analysis of meat quality-related traits in a large F2 intercross between Landrace and Korean native pigs. Genet Sel Evol. 2015;47:7 Epub 2015/04/19. 10.1186/s12711-014-0080-6 25888076 PMC4336478

[13] Choi I, Steibel JP, Bates RO, Raney NE, Rumph JM, Ernst CW. Identification of Carcass and Meat Quality QTL in an F(2) Duroc x Pietrain Pig Resource Population Using Different Least-Squares Analysis Models. Frontiers in genetics. 2011;2:18 Epub 2012/02/04. 10.3389/fgene.2011.00018 22303314 PMC3268573

[14] Ma J, Ren J, Guo Y, Duan Y, Ding N, Zhou L, et al Genome-wide identification of quantitative trait loci for carcass composition and meat quality in a large-scale White Duroc x Chinese Erhualian resource population. Anim Genet. 2009;40(5):637–47. 10.1111/j.1365-2052.2009.01892.x . 19397518

[15] Malek M, Dekkers JC, Lee HK, Baas TJ, Rothschild MF. A molecular genome scan analysis to identify chromosomal regions influencing economic traits in the pig. I. Growth and body composition. Mamm Genome. 2001;12(8):630–6. 10.1007/s003350020018 . 11471058

[16] Stachowiak M, Nowacka-Woszuk J, Szydlowski M, Switonski M. The ACACA and SREBF1 genes are promising markers for pig carcass and performance traits, but not for fatty acid content in the longissimus dorsi muscle and adipose tissue. Meat Science. 2013;95(1):64–71. 10.1016/j.meatsci.2013.04.021 23657179

[17] Thomsen H, Lee HK, Rothschild MF, Malek M, Dekkers JCM. Characterization of quantitative trait loci for growth and meat quality in a cross between commercial breeds of swine. Journal of Animal Science. 2004;82(8):2213–28. WOS:000222906600001. 10.2527/2004.8282213x 15318717

[18] Nagamine Y, Haley CS, Sewalem A, Visscher PM. Quantitative trait loci variation for growth and obesity between and within lines of pigs (Sus scrofa). Genetics. 2003;164(2):629–35. WOS:000183880000024. 12807783 10.1093/genetics/164.2.629 PMC1462585

[19] Andersson L. Genome-wide association analysis in domestic animals: a powerful approach for genetic dissection of trait loci. Genetica. 2009;136(2):341–9. 10.1007/s10709-008-9312-4 . 18704695

[20] Edwards DB, Ernst CW, Raney NE, Doumit ME, Hoge MD, Bates RO. Quantitative trait locus mapping in an F2 Duroc x Pietrain resource population: II. Carcass and meat quality traits. J Anim Sci. 2008;86(2):254–66. 10.2527/jas.2006-626 . 17965326

[21] Cherel P, Pires J, Glenisson J, Milan D, Iannuccelli N, Herault F, et al Joint analysis of quantitative trait loci and major-effect causative mutations affecting meat quality and carcass composition traits in pigs. BMC Genet. 2011;12:76 10.1186/1471-2156-12-76 21875434 PMC3175459

[22] Tabor HK, Risch NJ, Myers RM. Candidate-gene approaches for studying complex genetic traits: practical considerations. Nat Rev Genet. 2002;3(5):391–7. 10.1038/nrg796 . 11988764

[23] Qiao R, Gao J, Zhang Z, Li L, Xie X, Fan Y, et al Genome-wide association analyses reveal significant loci and strong candidate genes for growth and fatness traits in two pig populations. Genetics Selection Evolution. 2015;47(1):17. 10.1186/s12711-015-0089-5 PMC4358731 25885760

[24] Riquet J, Gilbert H, Servin B, Sanchez MP, Iannuccelli N, Billon Y, et al A locally congenic backcross design in pig: a new regional fine QTL mapping approach miming congenic strains used in mouse. Bmc Genetics. 2011;12 Artn 6 10.1186/1471-2156-12-6 WOS:000286728200001. 21235745 PMC3748014

[25] Wellcome Trust Case Control C. Genome-wide association study of 14,000 cases of seven common diseases and 3,000 shared controls. Nature. 2007;447(7145):661–78. 10.1038/nature05911 17554300 PMC2719288

[26] Daetwyler HD, Capitan A, Pausch H, Stothard P, van Binsbergen R, Brondum RF, et al Whole-genome sequencing of 234 bulls facilitates mapping of monogenic and complex traits in cattle. Nat Genet. 2014;46(8):858–65. 10.1038/ng.3034 . 25017103

[27] Xu SS, Gao L, Xie XL, Ren YL, Shen ZQ, Wang F, et al Genome-Wide Association Analyses Highlight the Potential for Different Genetic Mechanisms for Litter Size Among Sheep Breeds. Frontiers in genetics. 2018;9 ARTN 118 10.3389/fgene.2018.00118 WOS:000429604200001. 29692799 PMC5902979

[28] Ding R, Yang M, Wang X, Quan J, Zhuang Z, Zhou S, et al Genetic Architecture of Feeding Behavior and Feed Efficiency in a Duroc Pig Population. Frontiers in genetics. 2018;9:220 10.3389/fgene.2018.00220 29971093 PMC6018414

[29] Ma J, Yang J, Zhou L, Ren J, Liu X, Zhang H, et al A splice mutation in the PHKG1 gene causes high glycogen content and low meat quality in pig skeletal muscle. PLoS Genet. 2014;10(10):e1004710 10.1371/journal.pgen.1004710 25340394 PMC4207639

[30] Jung EJ, Park HB, Lee JB, Yoo CK, Kim BM, Kim HI, et al Genome-wide association analysis identifies quantitative trait loci for growth in a Landrace purebred population. Anim Genet. 2014;45(3):442–4. 10.1111/age.12117 . 24506094

[31] Visscher PM, Wray NR, Zhang Q, Sklar P, McCarthy MI, Brown MA, et al 10 Years of GWAS Discovery: Biology, Function, and Translation. Am J Hum Genet. 2017;101(1):5–22. 10.1016/j.ajhg.2017.06.005 28686856 PMC5501872

[32] Yu JM, Pressoir G, Briggs WH, Bi IV, Yamasaki M, Doebley JF, et al A unified mixed-model method for association mapping that accounts for multiple levels of relatedness. Nature Genetics. 2006;38(2):203–8. 10.1038/ng1702 WOS:000234953200015. 16380716

[33] Pharoah PDP, Tsai YY, Ramus SJ, Phelan CM, Goode EL, Lawrenson K, et al GWAS meta-analysis and replication identifies three new susceptibility loci for ovarian cancer. Nature Genetics. 2013;45(4):362–70. 10.1038/ng.2564 WOS:000316840600006. 23535730 PMC3693183

[34] Bouwman AC, Daetwyler HD, Chamberlain AJ, Ponce CH, Sargolzaei M, Schenkel FS, et al Meta-analysis of genome-wide association studies for cattle stature identifies common genes that regulate body size in mammals. Nature Genetics. 2018;50(3):362–+. 10.1038/s41588-018-0056-5 WOS:000427933400011. 29459679

[35] Wang Y, Ding XD, Tan Z, Ning C, Xing K, Yang T, et al Genome-Wide Association Study of Piglet Uniformity and Farrowing Interval. Frontiers in genetics. 2017;8 ARTN 194 10.3389/fgene.2017.00194 WOS:000416329800001. 29234349 PMC5712316

[36] Purcell S, Neale B, Todd-Brown K, Thomas L, Ferreira MA, Bender D, et al PLINK: a tool set for whole-genome association and population-based linkage analyses. Am J Hum Genet. 2007;81(3):559–75. Epub 2007/08/19. 10.1086/519795 17701901 PMC1950838

[37] Yang J, Lee SH, Goddard ME, Visscher PM. GCTA: a tool for genome-wide complex trait analysis. Am J Hum Genet. 2011;88(1):76–82. 10.1016/j.ajhg.2010.11.011 21167468 PMC3014363

[38] Price AL, Patterson NJ, Plenge RM, Weinblatt ME, Shadick NA, Reich D. Principal components analysis corrects for stratification in genome-wide association studies. Nat Genet. 2006;38(8):904–9. 10.1038/ng1847 . 16862161

[39] Zhou X, Stephens M. Genome-wide efficient mixed-model analysis for association studies. Nat Genet. 2012;44(7):821–4. 10.1038/ng.2310 22706312 PMC3386377

[40] Willer CJ, Li Y, Abecasis GR. METAL: fast and efficient meta-analysis of genomewide association scans. Bioinformatics. 2010;26(17):2190–1. 10.1093/bioinformatics/btq340 WOS:000281738900017. 20616382 PMC2922887

[41] Le TH, Christensen OF, Nielsen B, Sahana G. Genome-wide association study for conformation traits in three Danish pig breeds. Genet Sel Evol. 2017;49(1):12 10.1186/s12711-017-0289-2 28118822 PMC5259967

[42] Guo J, Sun C, Qu L, Shen M, Dou T, Ma M, et al Genetic architecture of bone quality variation in layer chickens revealed by a genome-wide association study. Sci Rep. 2017;7:45317 10.1038/srep45317 28383518 PMC5382839

[43] Barrett JC, Fry B, Maller J, Daly MJ. Haploview: analysis and visualization of LD and haplotype maps. Bioinformatics. 2005;21(2):263–5. 10.1093/bioinformatics/bth457 . 15297300

[44] Yang J, Ferreira T, Morris AP, Medland SE, Madden PA, Heath AC, et al Conditional and joint multiple-SNP analysis of GWAS summary statistics identifies additional variants influencing complex traits. Nat Genet. 2012;44(4):369–75, s1-3. Epub 2012/03/20. 10.1038/ng.2213 22426310 PMC3593158

[45] Xing K, Zhu F, Zhai LW, Chen SK, Tan Z, Sun YY, et al Identification of genes for controlling swine adipose deposition by integrating transcriptome, whole-genome resequencing, and quantitative trait loci data. Sci Rep-Uk. 2016;6 ARTN 23219 10.1038/srep23219 WOS:000372439900001. 26996612 PMC4800386

[46] Lee SH, Kim JM, Ryu YC, Ko KS. Effects of Morphological Characteristics of Muscle Fibers on Porcine Growth Performance and Pork Quality. Korean J Food Sci Anim Resour. 2016;36(5):583–93. 10.5851/kosfa.2016.36.5.583 27857533 PMC5112420

[47] Ryu YC, Rhee MS, Kim BC. Estimation of correlation coefficients between histological parameters and carcass traits of pig Longissimus dorsi muscle. Asian Austral J Anim. 2004;17(3):428–33. 10.5713/ajas.2004.428 WOS:000188505500023.

[48] Xin L, Wang LG, Jing L, Hua Y, Zhao KB, Na LI, et al Genome-Wide Association Study for Certain Carcass Traits and Organ Weights in a Large White×Minzhu Intercross Porcine Population. Journal of Integrative Agriculture. 2014;13(12):2721–30.

[49] Ma J, Yang J, Zhou L, Zhang Z, Ma H, Xie X, et al Genome-wide association study of meat quality traits in a White DurocxErhualian F2 intercross and Chinese Sutai pigs. PLoS One. 2013;8(5):e64047 10.1371/journal.pone.0064047 23724019 PMC3665833

[50] Liu X, Huang M, Fan B, Buckler ES, Zhang Z. Iterative Usage of Fixed and Random Effect Models for Powerful and Efficient Genome-Wide Association Studies. PLoS Genet. 2016;12(2):e1005767 10.1371/journal.pgen.1005767 26828793 PMC4734661

[51] Vilhjalmsson BJ, Nordborg M. The nature of confounding in genome-wide association studies. Nat Rev Genet. 2013;14(1):1–2. 10.1038/nrg3382 . 23165185

[52] Sham PC, Purcell SM. Statistical power and significance testing in large-scale genetic studies. Nature Reviews Genetics. 2014;15(5):335–46. 10.1038/nrg3706 24739678

[53] Wei HK, Zhou YF, Jiang SZ, Huang FR, Peng J, Jiang SW. Transcriptional response of porcine skeletal muscle to feeding a linseed-enriched diet to growing pigs. J Anim Sci Biotechno. 2016;7 ARTN 6 10.1186/s40104-016-0064-1 WOS:000369590500001. 26862397 PMC4746901

[54] Damon M, Wyszynska-Koko J, Vincent A, Herault F, Lebret B. Comparison of Muscle Transcriptome between Pigs with Divergent Meat Quality Phenotypes Identifies Genes Related to Muscle Metabolism and Structure. Plos One. 2012;7(3). ARTN e33763 10.1371/journal.pone.0033763 WOS:000303857100047. 22470472 PMC3312351

[55] Steibel JP, Bates RO, Rosa GJM, Tempelman RJ, Rilington VD, Ragavendran A, et al Genome-Wide Linkage Analysis of Global Gene Expression in Loin Muscle Tissue Identifies Candidate Genes in Pigs. Plos One. 2011;6(2). ARTN e16766 10.1371/journal.pone.0016766 WOS:000287077600023. 21346809 PMC3035619

[56] Yang X, Khosravi-Far R, Chang HY, Baltimore D. Daxx, a Novel Fas-Binding Protein That Activates JNK and Apoptosis. Cell. 1997;89(7):1067–76. 9215629 10.1016/s0092-8674(00)80294-9 PMC2989411

[57] Choi JR, Kim JY, Park IH, Huh JH, Kim KW, Cha SK, et al Serum Fibroblast Growth Factor 21 and New-Onset Metabolic Syndrome: KoGES-ARIRANG Study. Yonsei Medical Journal. 2018;59(2):287–93. 10.3349/ymj.2018.59.2.287 29436198 PMC5823832

[58] Zhang X, Yeung DCY, Karpisek M, Stejskal D, Zhou ZG, Liu F, et al Serum FGF21 levels are increased in obesity and are independently associated with the metabolic syndrome in humans. Diabetes. 2008;57(5):1246–53. 10.2337/db07-1476 WOS:000255628700014. 18252893

[59] Ayuso M, Fernandez A, Nunez Y, Benitez R, Isabel B, Barragan C, et al Comparative Analysis of Muscle Transcriptome between Pig Genotypes Identifies Genes and Regulatory Mechanisms Associated to Growth, Fatness and Metabolism. Plos One. 2015;10(12). ARTN e0145162 10.1371/journal.pone.0145162 WOS:000367092500025. 26695515 PMC4687939

[60] Potthoff MJ, Inagaki T, Satapati S, Ding X, He T, Goetz R, et al FGF21 induces PGC-1alpha and regulates carbohydrate and fatty acid metabolism during the adaptive starvation response. Proceedings of the National Academy of Sciences of the United States of America. 2009;106(26):10853–8. 10.1073/pnas.0904187106 19541642 PMC2705613

[61] Robinson MM, Yatherajam G, Ranallo RT, Bric A, Paule MR, Stargell LA. Mapping and functional characterization of the TAF11 interaction with TFIIA. Mol Cell Biol. 2005;25(3):945–57. 10.1128/MCB.25.3.945-957.2005 15657423 PMC543996

[62] Goumidi L, Cottel D, Dallongeville J, Amouyel P, Meirhaeghe A. Effects of established BMI-associated loci on obesity-related traits in a French representative population sample. BMC Genet. 2014;15:62 10.1186/1471-2156-15-62 24885863 PMC4035696

[63] Williams MJ, Eriksson A, Shaik M, Voisin S, Yamskova O, Paulsson J, et al The Obesity-Linked Gene Nudt3 Drosophila Homolog Aps Is Associated With Insulin Signaling. Mol Endocrinol. 2015;29(9):1303–19. 10.1210/ME.2015-1077 WOS:000365272700008. 26168034 PMC5414682

[64] Shears SB. Diphosphoinositol polyphosphates: metabolic messengers? Molecular Pharmacology. 2009;76(2):236 10.1124/mol.109.055897 19439500 PMC2713120

[65] Baranski TJ, Kraja AT, Fink JL, Feitosa M, Lenzini PA, Borecki IB, et al A high throughput, functional screen of human Body Mass Index GWAS loci using tissue-specific RNAi Drosophila melanogaster crosses. Plos Genetics. 2018;14(4). ARTN e100722210.1371/journal.pgen.1007222. WOS:000431115700003. 10.1371/journal.pgen.1007222 PMC5897035 29608557

[66] Sun H, Wang Z, Zhang Z, Xiao Q, Mawed S, Xu Z, et al Genomic signatures reveal selection of characteristics within and between Meishan pig populations. Anim Genet. 2018;49(2):119–26. Epub 2018/03/07. 10.1111/age.12642 . 29508928

[67] Wang L, Zhang L, Yan H, Liu X, Li N, Liang J, et al Genome-wide association studies identify the loci for 5 exterior traits in a Large White x Minzhu pig population. PLoS One. 2014;9(8):e103766 Epub 2014/08/05. 10.1371/journal.pone.0103766 25090094 PMC4121205

[68] Jiang Y, Tang S, Wang C, Wang Y, Qin Y, Wang Y, et al A genome-wide association study of growth and fatness traits in two pig populations with different genetic backgrounds. J Anim Sci. 2018;96(3):806–16. 10.1093/jas/skx038 29528397 PMC6093525

[69] Guo YM, Huang YX, Hou LJ, Ma JW, Chen CY, Ai HS, et al Genome-wide detection of genetic markers associated with growth and fatness in four pig populations using four approaches. Genetics Selection Evolution. 2017;49 ARTN 21 10.1186/s12711-017-0295-4 WOS:000394716100001. 28196480 PMC5307927

