# Supplementary figures and images for: Meta-analysis of genome-wide association studies for loin muscle area and loin muscle depth in two Duroc pig populations
Source: PLoS One. 2019 Jun 12;14(6):e0218263. doi: 10.1371/journal.pone.0218263 (PMC6561594; doi:10.1371/journal.pone.0218263)

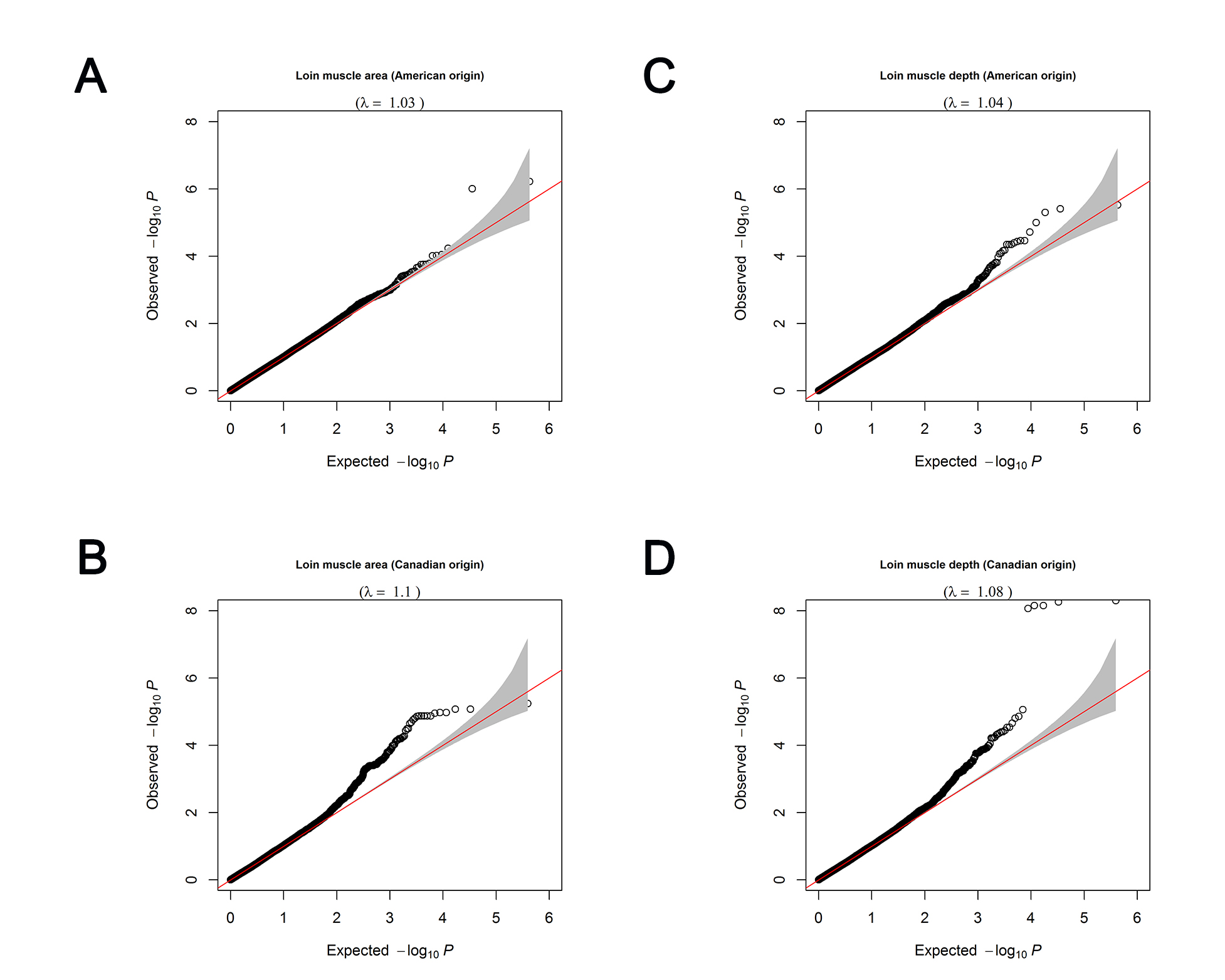

Supplement: S1 Fig — Q–Q plots show the observed versus expected negative log10 P-values. The left panel of the figure shows Q–Q plots for LMA in (A) the American original population and (B) the Canadian original population, respectively. The right panel of the figure shows Q–Q plots for LMD in (C) the American original population and (D) the Canadian original population, respectively. (TIF) [file pone.0218263.s001.tif]
