# Supplementary material for: Meta-analysis of genome-wide association studies for loin muscle area and loin muscle depth in two Duroc pig populations
Source: PLoS One. 2019 Jun 12;14(6):e0218263. doi: 10.1371/journal.pone.0218263 (PMC6561594; doi:10.1371/journal.pone.0218263)
Supplement: S1 Table — (DOCX) [file pone.0218263.s002.docx]

**Supplementary Material**

**Meta-analysis of genome-wide association studies for loin muscle area and loin muscle depth in two Duroc pig populations**

Zhanwei Zhuang^1$^, Shaoyun Li^1$^, Rongrong Ding^1^, Ming Yang^2^, Enqin Zheng^1^, Huaqiang Yang^1^, Ting Gu^1^, Zheng Xu^1^, Gengyuan Cai^1^, Zhenfang Wu^1,2^_*_, Jie Yang ^1^_*_

1 College of Animal Science and National Engineering Research Center for Breeding Swine Industry, South China Agricultural University, Guangdong, P.R. China.

2 National Engineering Research Center for Breeding Swine Industry, Guangdong Wens Foodstuffs Group Co., Ltd, Guangdong, P.R. China.

^$^These authors contributed equally to this work.

^*^Correspondence and requests for materials should be addressed to J.Y. (email: jieyang2012@hotmail.com) or Z.W. (email: wzfeamil@163.com)

**S1 Table. Significant SNPs and associated genes for LMA and LMD in the meta-analysis**

| Trait | SSC^1^ | SNP ID | Position(bp)^2^ | *P*_(LMA)_value^3^ | *P*_(LMD)_value^4^ | Candidate gene | Distance/bp^5^ |
| --- | --- | --- | --- | --- | --- | --- | --- |
| LMA | 7 | MARC0059955 | 22279830 | 7.19E-06 | - | *GPX5* | -9284 |
| LMA | 7 | DIAS0000302 | 24165493 | 1.61E-05 | - | *ATF6B* | within |
| LMA | 7 | WU_10.2_7_31453405 | 27011172 | 2.77E-06 | - | *LRRC1* | 21502 |
| LMA | 7 | ALGA0039950 | 27057971 | 3.18E-06 | - | *KLHL31* | -4073 |
| LMA | 7 | ASGA0032215 | 27107091 | 1.32E-05 | - | *KLHL31* | 974 |
| LMA | 7 | H3GA0020592 | 27221785 | 3.27E-06 | - | *GCLC* | within |
| LMA | 7 | ALGA0039974 | 27333265 | 6.68E-06 | - | *KHDRBS2* | -56609 |
| LMA | 7 | H3GA0020604 | 27394424 | 2.21E-05 | - | *KHDRBS2* | within |
| LMA | 7 | ASGA0100868 | 27487944 | 1.89E-05 | - | *KHDRBS2* | within |
| LMA | 7 | ALGA0115197 | 27504926 | 1.50E-05 | - | *KHDRBS2* | within |
| LMA | 7 | MARC0098266 | 27519266 | 1.46E-05 | - | *KHDRBS2* | within |
| LMA | 7 | ASGA0032245 | 27562670 | 2.01E-05 | - | *KHDRBS2* | within |
| LMA | 7 | ALGA0040000 | 27707775 | 2.35E-05 | - | *KHDRBS2* | within |
| LMA | 7 | ASGA0032254 | 27739781 | 1.51E-05 | - | *KHDRBS2* | within |
| LMA | 7 | ASGA0032255 | 27765368 | 1.45E-05 | - | *KHDRBS2* | within |
| LMA | 7 | MARC0050171 | 27789031 | 1.40E-05 | - | *KHDRBS2* | within |
| LMA | 7 | DRGA0007457 | 27809797 | 2.05E-05 | - | *KHDRBS2* | within |
| LMA | 7 | DRGA0007459 | 27840937 | 2.24E-05 | - | *KHDRBS2* | within |
| LMA | 7 | INRA0024655 | 27886752 | 4.56E-06 | - | *KHDRBS2* | within |
| LMA | 7 | ASGA0032257 | 27905935 | 1.68E-06 | - | *KHDRBS2* | 9866 |
| LMA | 7 | ASGA0032262 | 27963262 | 2.49E-06 | - | *KHDRBS2* | 67193 |
| LMA | 7 | WU_10.2_7_32423250 | 27996419 | 2.12E-06 | - | *KHDRBS2* | 100350 |
| LMA | 7 | DRGA0007462 | 28072350 | 2.12E-06 | - | *KHDRBS2* | 176281 |
| LMA | 7 | MARC0061142 | 30716800 | 2.46E-06 | - | *SNRPC* | within |
| LMA | 16 | DRGA0016090 | 33372038 | 2.19E-06 | - | *ARL15* | within |
| LMA | 16 | ASGA0072998 | 33589982 | 1.31E-06 | - | *ARL15* | 24383 |
| LMA | 16 | ALGA0090190 | 33515233 | **8.57E-07** | - | *ARL15* | within |
| LMA | 16 | ALGA0090184 | 33467573 | **9.17E-07** | - | *ARL15* | within |
| LMA | 16 | MARC0103451 | 33493718 | 1.26E-06 | - | *ARL15* | within |
| LMA | 16 | MARC0074818 | 33559933 | 2.18E-06 | - | *ARL15* | within |
| LMA | 16 | ASGA0073002 | 33636702 | **5.71E-07** | - | *ARL15* | 71103 |
| LMA/LMD | 6 | Hal_2/Hal | 47357966 | **6.05E-07** | 3.88E-06 | *RASGRP4* | 24726 |
| LMA/LMD | 6 | 12784654 | 54079560 | **9.84E-07** | 4.97E-06 | *FGF21* | -3046 |
| LMA/LMD | 7 | ALGA0039474 | 22126764 | 1.22E-05 | 5.56E-06 | *ZSCAN26* | within |
| LMA/LMD | 7 | ALGA0039480 | 22184943 | 4.79E-06 | 1.41E-05 | *ZSCAN31* | 3767 |
| LMA/LMD | 7 | ASGA0031873 | 22205854 | 6.82E-06 | 4.04E-06 | *ZSCAN12* | within |
| LMA/LMD | 7 | ASGA0031928 | 22682744 | 1.85E-06 | 3.08E-06 | *TRIM31* | 1711 |
| LMA/LMD | 7 | MARC0010879 | 27386432 | 7.73E-06 | 2.41E-05 | *KHDRBS2* | -3442 |
| LMA/LMD | 7 | SIRI0000698 | 27549577 | 5.43E-06 | 1.63E-05 | *KHDRBS2* | within |
| LMA/LMD | 7 | ASGA0032250 | 27652363 | 7.96E-06 | 1.91E-05 | *KHDRBS2* | within |
| LMA/LMD | 7 | WU_10.2_7_35213538 | 30536003 | 1.13E-05 | **3.77E-07** | *SPDEF* | 11732 |
| LMA/LMD | 7 | ASGA0032562 | 30786798 | 1.50E-05 | **8.47E-07** | *TAF11* | -13167 |
| LMA/LMD | 7 | WU_10.2_7_35659198 | 30885616 | 5.60E-06 | **2.69E-07** | *ANKS1A* | within |
| LMA/LMD | 7 | ALGA0040298 | 30893735 | 3.13E-06 | **1.11E-07** | *ANKS1A* | within |
| LMA/LMD | 7 | INRA0027601 | 96278617 | 7.84E-06 | **6.62E-07** | *DCAF4* | -10186 |
| LMA/LMD | 7 | Affx-114687136 | 97568284 | 1.35E-06 | **5.87E-08** | *ABCD4* | within |
| LMA/LMD | 7 | Affx-114892585 | 97575068 | 2.48E-06 | **7.27E-08** | *ABCD4* | within |
| LMA/LMD | 7 | WU_10.2_7_103232787 | 97584287 | 2.96E-06 | **5.55E-08** | *ABCD4* | within |
| LMA/LMD | 7 | WU_10.2_7_103460706 | 97617907 | 2.48E-06 | **7.27E-08** | *VRTN* | within |
| LMA/LMD | 7 | MARC0038565 | 97652632 | 2.46E-06 | **1.73E-07** | *SYNDIG1L* | -4101 |
| LMA/LMD | 7 | ALGA0040260 | 30342161 | **2.03E-07** | **8.63E-11** | *NUDT3* | -1092 |
| LMA/LMD | 7 | ALGA0040263 | 30356985 | **2.32E-07** | **9.28E-11** | *NUDT3* | within |
| LMA/LMD | 7 | ASGA0032536 | 30476054 | **1.30E-07** | **3.88E-11** | *PACSIN1* | within |
| LMA/LMD | 7 | ASGA0032526 | 30497305 | **3.59E-07** | **1.36E-10** | *PACSIN1* | within |
| LMA/LMD | 7 | INRA0024788 | 30317219 | **1.37E-07** | **3.45E-11** | *ENSSSCG00000032242* | -3195 |
| LMA/LMD | 7 | DRGA0007345 | 21886880 | **5.58E-07** | 1.07E-07 | *ENSSSCG00000032646* | 474 |
| LMD | 7 | H3GA0020268 | 20399615 | - | 2.31E-05 | *SCGN* | within |
| LMD | 7 | ALGA0039400 | 21166373 | - | 3.47E-06 | *ZNF322* | 22986 |
| LMD | 7 | ALGA0039477 | 22152108 | - | 4.91E-06 | *PGBD1* | within |
| LMD | 7 | H3GA0020334 | 22172927 | - | 6.37E-06 | *ZSCAN31* | -541 |
| LMD | 7 | M1GA0009777 | 23985825 | - | 1.70E-05 | *EHMT2* | within |
| LMD | 7 | MARC0069646 | 29599648 | - | 1.83E-05 | *VPS52* | -23898 |
| LMD | 7 | ASGA0032422 | 29685247 | - | 2.10E-05 | *DAXX* | 4137 |
| LMD | 7 | WU_10.2_7_34313764 | 29781372 | - | 1.73E-05 | *BAK1* | -25639 |
| LMD | 7 | M1GA0009960 | 30083247 | - | 1.31E-05 | *MLN* | 70119 |
| LMD | 7 | ALGA0040291 | 30821002 | - | 1.51E-06 | *ANKS1A* | within |
| LMD | 7 | MARC0061142 | 30716800 | - | 3.51E-08 | *SNRPC* | within |
| LMD | 18 | WU_10.2_18_58542037 | 53382393 | - | 1.27E-05 | *INHBA* | 477180 |

^1^*Sus scrofa* chromosome. ^2^SNP position in Ensembl. ^3^*P*_(LMA)_ value refers to the *P* value of significant SNP in meta-analysis for LMA; The bold data in this column represent the significant SNP surpass the genome-wide significant threshold; - represent the SNP not surpass the minimum threshold. ^4^P_(LMD)_ value refers to the *P* value of significant SNP in meta-analysis for LMD; The bold data in this column represent the significant SNP surpass the genome-wide significant threshold. ^5^The location of SNP in upstream/downstream of the nearest gene.
