# Supplementary material for: Meta-analysis of genome-wide association studies for loin muscle area and loin muscle depth in two Duroc pig populations
Source: PLoS One. 2019 Jun 12;14(6):e0218263. doi: 10.1371/journal.pone.0218263 (PMC6561594; doi:10.1371/journal.pone.0218263)
Supplement: S2 Table — (DOCX) [file pone.0218263.s003.docx]

**Supplementary Material**

**Meta-analysis of genome-wide association studies for loin muscle area and loin muscle depth in two Duroc pig populations**

Zhanwei Zhuang^1$^, Shaoyun Li^1$^, Rongrong Ding^1^, Ming Yang^2^, Enqin Zheng^1^, Huaqiang Yang^1^, Ting Gu^1^, Zheng Xu^1^, Gengyuan Cai^1^, Zhenfang Wu^1,2^_*_, Jie Yang ^1^_*_

1 College of Animal Science and National Engineering Research Center for Breeding Swine Industry, South China Agricultural University, Guangdong, P.R. China.

2 National Engineering Research Center for Breeding Swine Industry, Guangdong Wens Foodstuffs Group Co., Ltd, Guangdong, P.R. China.

^$^These authors contributed equally to this work.

^*^Correspondence and requests for materials should be addressed to J.Y. (email: jieyang2012@hotmail.com) or Z.W. (email: wzfeamil@163.com)

**S2 Table. Comparative mapping of tag SNPs with previous QTLs reported in the pig QTL database (as of September 26, 2018) and previous GWAS results.**

| **SSC^1^** | **SNP ID** | **Location (bp)^2^** | **QTL location range^3^** | **QTLID^4^** | **Related QTL^5^** | **Trait** |
| --- | --- | --- | --- | --- | --- | --- |
| 6 | Hal_2 | 47357966 | 6:34464154-66533116 | 38053 | Loin muscle area | LMA/LMD |
| 6 | 12784654 | 54079560 | 6:34464154-66533116 | 38053 | Loin muscle area | LMA/LMD |
| 7 | H3GA0020268 | 20399615 | 7:7434494-124300676 | 38092 | Loin muscle area | LMD |
| 7 | ALGA0039400 | 21166373 | 7:7434494-124300676 | 38092 | Loin muscle area | LMD |
| 7 | DRGA0007345 | 21886880 | 7:7434494-124300676 | 38092 | Loin muscle area | LMA/LMD |
| 7 | ALGA0039447 | 21940478 | 7:7434494-124300676 | 38092 | Loin muscle area | LMD |
| 7 | ALGA0039474 | 22126764 | 7:7434494-124300676 | 38092 | Loin muscle area | LMA/LMD |
| 7 | ALGA0039477 | 22152108 | 7:7434494-124300676 | 38092 | Loin muscle area | LMD |
| 7 | H3GA0020334 | 22172927 | 7:7434494-124300676 | 38092 | Loin muscle area | LMD |
| 7 | ALGA0039480 | 22184943 | 7:7434494-124300676 | 38092 | Loin muscle area | LMA/LMD |
| 7 | ASGA0031873 | 22205854 | 7:7434494-124300676 | 38092 | Loin muscle area | LMA/LMD |
| 7 | MARC0059955 | 22279830 | 7:7434494-124300676 | 38092 | Loin muscle area | LMA |
| 7 | ASGA0031928 | 22682744 | 7:7434494-124300676 | 38092 | Loin muscle area | LMA/LMD |
| 7 | M1GA0009777 | 23985825 | 7:7434494-124300676 | 38092 | Loin muscle area | LMD |
| 7 | DIAS0000302 | 24165493 | 7:7434494-124300676 | 38092 | Loin muscle area | LMA |
| 7 | M1GA0027226 | 26125115 | 7:7434494-124300676 | 38092 | Loin muscle area | LMD |
| 7 | ALGA0039866 | 26451150 | 7:7434494-124300676 | 38092 | Loin muscle area | LMD |
| 7 | WU_10.2_7_31453405 | 27011172 | 7:7434494-124300676 | 38092 | Loin muscle area | LMA |
| 7 | ALGA0039950 | 27057971 | 7:7434494-124300676 | 38092 | Loin muscle area | LMA |
| 7 | ASGA0032215 | 27107091 | 7:7434494-124300676 | 38092 | Loin muscle area | LMA |
| 7 | H3GA0020592 | 27221785 | 7:7434494-124300676 | 38092 | Loin muscle area | LMA |
| 7 | ALGA0039974 | 27333265 | 7:7434494-124300676 | 38092 | Loin muscle area | LMA |
| 7 | MARC0010879 | 27386432 | 7:7434494-124300676 | 38092 | Loin muscle area | LMA/LMD |
| 7 | H3GA0020604 | 27394424 | 7:7434494-124300676 | 38092 | Loin muscle area | LMA |
| 7 | ASGA0100868 | 27487944 | 7:7434494-124300676 | 38092 | Loin muscle area | LMA |
| 7 | ALGA0115197 | 27504926 | 7:7434494-124300676 | 38092 | Loin muscle area | LMA |
| 7 | MARC0098266 | 27519266 | 7:7434494-124300676 | 38092 | Loin muscle area | LMA |
| 7 | SIRI0000698 | 27549577 | 7:7434494-124300676 | 38092 | Loin muscle area | LMA/LMD |
| 7 | ASGA0032245 | 27562670 | 7:7434494-124300676 | 38092 | Loin muscle area | LMA |
| 7 | ASGA0032250 | 27652363 | 7:7434494-124300676 | 38092 | Loin muscle area | LMA/LMD |
| 7 | ALGA0040000 | 27707775 | 7:7434494-124300676 | 38092 | Loin muscle area | LMA |
| 7 | ASGA0032254 | 27739781 | 7:7434494-124300676 | 38092 | Loin muscle area | LMA |
| 7 | ASGA0032255 | 27765368 | 7:7434494-124300676 | 38092 | Loin muscle area | LMA |
| 7 | MARC0050171 | 27789031 | 7:7434494-124300676 | 38092 | Loin muscle area | LMA |
| 7 | DRGA0007457 | 27809797 | 7:7434494-124300676 | 38092 | Loin muscle area | LMA |
| 7 | DRGA0007459 | 27840937 | 7:7434494-124300676 | 38092 | Loin muscle area | LMA |
| 7 | INRA0024655 | 27886752 | 7:7434494-124300676 | 38092 | Loin muscle area | LMA |
| 7 | ASGA0032257 | 27905935 | 7:7434494-124300676 | 38092 | Loin muscle area | LMA |
| 7 | ASGA0032262 | 27963262 | 7:7434494-124300676 | 38092 | Loin muscle area | LMA |
| 7 | WU_10.2_7_32423250 | 27996419 | 7:7434494-124300676 | 38092 | Loin muscle area | LMA |
| 7 | DRGA0007462 | 28072350 | 7:7434494-124300676 | 38092 | Loin muscle area | LMA |
| 7 | MARC0069646 | 29599648 | 7:7434494-124300676 | 38092 | Loin muscle area | LMD |
| 7 | ASGA0032422 | 29685247 | 7:7434494-124300676 | 38092 | Loin muscle area | LMD |
| 7 | WU_10.2_7_34313764 | 29781372 | 7:7434494-124300676 | 38092 | Loin muscle area | LMD |
| 7 | M1GA0009960 | 30083247 | 7:7434494-124300676 | 38092 | Loin muscle area | LMD |
| 7 | INRA0024788 | 30317219 | 7:7434494-124300676 | 38092 | Loin muscle area | LMA/LMD |
| 7 | ALGA0040260 | 30342161 | 7:7434494-124300676 | 38092 | Loin muscle area | LMA/LMD |
| 7 | ALGA0040263 | 30356985 | 7:7434494-124300676 | 38092 | Loin muscle area | LMA/LMD |
| 7 | ASGA0032536 | 30476054 | 7:7434494-124300676 | 38092 | Loin muscle area | LMA/LMD |
| 7 | ASGA0032526 | 30497305 | 7:7434494-124300676 | 38092 | Loin muscle area | LMA/LMD |
| 7 | WU_10.2_7_35213538 | 30536003 | 7:7434494-124300676 | 38092 | Loin muscle area | LMA/LMD |
| 7 | MARC0061142 | 30716800 | 7:7434494-124300676 | 38092 | Loin muscle area | LMD |
| 7 | ASGA0032562 | 30786798 | 7:7434494-124300676 | 38092 | Loin muscle area | LMA/LMD |
| 7 | ALGA0040291 | 30821002 | 7:7434494-124300676 | 38092 | Loin muscle area | LMD |
| 7 | WU_10.2_7_35659198 | 30885616 | 7:7434494-124300676 | 38092 | Loin muscle area | LMA/LMD |
| 7 | ALGA0040298 | 30893735 | 7:7434494-124300676 | 38092 | Loin muscle area | LMA/LMD |
| 7 | INRA0027601 | 96278617 | 7:7434494-124300676 | 38092 | Loin muscle area | LMA/LMD |
| 7 | Affx-114687136 | 97568284 | 7:7434494-124300676 | 38092 | Loin muscle area | LMA/LMD |
| 7 | Affx-114892585 | 97575068 | 7:7434494-124300676 | 38092 | Loin muscle area | LMA/LMD |
| 7 | WU_10.2_7_103232787 | 97584287 | 7:7434494-124300676 | 38092 | Loin muscle area | LMA/LMD |
| 7 | WU_10.2_7_103460706 | 97617907 | 7:7434494-124300676 | 38092 | Loin muscle area | LMA/LMD |
| 7 | MARC0038565 | 97652632 | 7:7434494-124300676 | 38092 | Loin muscle area | LMA/LMD |
| 12 | ALGA0065784 | 26376192 | 12:23672762-60040247 | 3085 | Loin muscle area | LMD |
| 16 | DRGA0016090 | 33372038 | 16:22335189-42435187 | 21423 | Loin muscle area | LMA |
| 16 | ALGA0090184 | 33467573 | 16:22335189-42435187 | 21423 | Loin muscle area | LMA |
| 16 | MARC0103451 | 33493718 | 16:22335189-42435187 | 21423 | Loin muscle area | LMA |
| 16 | ALGA0090190 | 33515233 | 16:22335189-42435187 | 21423 | Loin muscle area | LMA |
| 16 | MARC0074818 | 33559933 | 16:22335189-42435187 | 21423 | Loin muscle area | LMA |
| 16 | ASGA0072998 | 33589982 | 16:22335189-42435187 | 21423 | Loin muscle area | LMA |
| 16 | ASGA0073002 | 33636702 | 16:22335189-42435187 | 21423 | Loin muscle area | LMA |
| 16 | WU_10.2_16_35829257 | 33757844 | 16:22335189-42435187 | 21423 | Loin muscle area | LMA |
| 16 | ALGA0090242 | 34031872 | 16:22335189-42435187 | 21423 | Loin muscle area | LMA |
| 16 | ALGA0090276 | 34334858 | 16:22335189-42435187 | 21423 | Loin muscle area | LMA |
| 16 | ALGA0090273 | 34364519 | 16:22335189-42435187 | 21423 | Loin muscle area | LMA |
| 18 | WU_10.2_18_58542037 | 53382393 | 18:52307963-77675048 | 12539 | Backfat at last rib | LMD |

^1^Sus scrofa chromosome. ^2^SNP position in Ensembl. ^3^Location range of the mapped QTL in the QTL database. ^4^Identity of QTL in the pig QTL database or published literature. ^5^The name of mapped QTL in the database (https://www.animalgenome.org/cgi-bin/QTLdb/SS/index).
